# Speed-Accuracy Tradeoffs in Brain and Behavior: Testing the Independence of P300 and N400 Related Processes in Behavioral Responses to Sentence Categorization

**DOI:** 10.3389/fnhum.2019.00285

**Published:** 2019-08-27

**Authors:** Phillip M. Alday, Franziska Kretzschmar

**Affiliations:** ^1^Max Planck Institute for Psycholinguistics, Nijmegen, Netherlands; ^2^CRC 1252 “Prominence in Language”, University of Cologne, Cologne, Germany; ^3^Institute of German Language and Literature I, University of Cologne, Cologne, Germany

**Keywords:** N400, P300, mixed-effects modeling, SAT, sentence processing, predictive processing

## Abstract

Although the N400 was originally discovered in a paradigm designed to elicit a P300 (Kutas and Hillyard, 1980), its relationship with the P300 and how both overlapping event-related potentials (ERPs) determine behavioral profiles is still elusive. Here we conducted an ERP (*N* = 20) and a multiple-response speed-accuracy tradeoff (SAT) experiment (*N* = 16) on distinct participant samples using an antonym paradigm (*The opposite of black is white/nice/yellow* with acceptability judgment). We hypothesized that SAT profiles incorporate processes of task-related decision-making (P300) and stimulus-related expectation violation (N400). We replicated previous ERP results (Roehm et al., 2007): in the correct condition (*white*), the expected target elicits a P300, while both expectation violations engender an N400 [reduced for related (*yellow*) vs. unrelated targets (*nice*)]. Using multivariate Bayesian mixed-effects models, we modeled the P300 and N400 responses simultaneously and found that correlation between residuals and subject-level random effects of each response window was minimal, suggesting that the components are largely independent. For the SAT data, we found that antonyms and unrelated targets had a similar slope (rate of increase in accuracy over time) and an asymptote at ceiling, while related targets showed both a lower slope and a lower asymptote, reaching only approximately 80% accuracy. Using a GLMM-based approach (Davidson and Martin, 2013), we modeled these dynamics using response time and condition as predictors. Replacing the predictor for condition with the averaged P300 and N400 amplitudes from the ERP experiment, we achieved identical model performance. We then examined the piecewise contribution of the P300 and N400 amplitudes with partial effects (see Hohenstein and Kliegl, 2015). Unsurprisingly, the P300 amplitude was the strongest contributor to the SAT-curve in the antonym condition and the N400 was the strongest contributor in the unrelated condition. In brief, this is the first demonstration of how overlapping ERP responses in one sample of participants predict behavioral SAT profiles of another sample. The P300 and N400 reflect two independent but interacting processes and the competition between these processes is reflected differently in behavioral parameters of speed and accuracy.

## Introduction

Human cognition can be conceived of as a dynamic, hierarchically organized system of decision-making or categorization that accumulates evidence for (alternative) categories as new incoming sensory information is processed across time, and translates the outcome of this categorization to appropriate action once a decision threshold has been reached (Gold and Shadlen, 2007; Kelly and O’Connell, 2015).

Language is no exception to this: linguistic categorization is a dynamic process in which evidence from stimulus properties from lower to higher linguistic levels is accumulated across time, shaped by both stimulus-induced (exogeneous) processes as well as decision-related (endogenous) processes. Associating sounds to phonemes, phoneme sequences to words and words to larger sentences are (somewhat simplified) examples for how humans categorize spoken linguistic input to compute the meaning of an utterance and subsequently plan an appropriate response. Importantly, predictive processing has been identified as a major (endogenous) mechanism in language comprehension that facilitates linguistic categorization in terms of processing speed and accuracy, as predictable linguistic units are processed faster and comprehended with fewer errors than unpredictable ones.

Our motivation for the current study is the observation that a fairly high number of studies on word recognition in isolation or in context report mixed evidence for effects of semantic prediction and relatedness/priming when comparing electrophysiological signatures such as event-related potentials (ERPs) with behavioral measures such as error rates (ER) and reaction time (RT). We restrict ourselves to studies that investigated how words are categorized as belonging to a certain semantic category by focusing on N400 and P300 ERPs in response to contextual predictability and semantic relatedness/priming with various experimental tasks (i.e., acceptability judgment, semantic categorization or comprehension tasks). As we will outline in more detail below, these studies reported a mixture of converging (i.e., identical effect directions of increases/decreases in ERP amplitudes, RT and ER) and diverging effects of these variables in the electrophysiological and behavioral data, a pattern that eludes a fully systematic explanation. More specifically, we conjecture that contextual predictability and semantic relatedness may impact ERPs differently than behavioral measures and that this interaction is additionally modulated by methodical complications. That is, cross-method divergence results in part from two well-known complications, namely that N400 and P300 overlap in time and scalp topography despite their different cognitive functions, and that standard RT and/or ER measures rely on a single data point insensitive to the dynamics of categorization. This makes it difficult to unify, across electrophysiological and behavioral measures, effects of contextual predictability and semantic relatedness in signatures of stimulus processing and categorization at the word or sentence level.

The present article aims at presenting a novel cross-method approach to address this issue, and thereby to increase the validity of cross-method inferences on brain-behavior links or the perception-action loop in language processing—i.e., the time-course from neuronal processing (perception and categorization) to behavioral output (action). We specifically propose that the above complications may be overcome with time-sensitive behavioral measures such as the speed-accuracy trade-off (SAT) paradigm (Wickelgren, 1977) replacing standard RT measures and capturing decision dynamics more precisely, and with cross-method statistical modeling using mixed-effects models.

The N400 and the P300 are probably among the most intensively used ERP components to study language processing in humans and it is therefore not surprising that the range of their functional definitions varies tremendously. The following is thus not meant as a review of the extensive N400 and P300 literature but is highly selective in focusing on ERP-behavior relationships. The N400 is a negative-going deflection in the scalp-recorded EEG that peaks about 400 ms after the onset of a meaningful stimulus, showing a posterior maximum (Kutas and Federmeier, 2000, 2011). In particular for word recognition, the N400 has been found in response to words embedded in word lists, sentences and stories as well as in all modalities of language input (e.g., Kutas et al., 1987; Holcomb and Neville, 1990; Federmeier and Kutas, 1999; Alday et al., 2017). N400 amplitude is sensitive to a range of (broadly defined) semantic variables such as lexical frequency, contextual predictability, semantic relatedness/association, lexicality or orthographic neighborhood density (Kutas and Federmeier, 2000, 2011; Laszlo and Federmeier, 2009), but has also been found for processing at the syntax-semantics interface (e.g., Haupt et al., 2008; Bornkessel-Schlesewsky et al., 2011; Bourguignon et al., 2012) and discourse (e.g., van Berkum et al., 1999; Burkhardt, 2006). Predictability, including semantic priming as a subtype, has been found in particular to reduce N400 amplitude (Kutas and Federmeier, 2000; Federmeier, 2007; Van Petten and Luka, 2012). Building on this, it has been posited that amplitude increases to unpredictable input reflect either varying pre-activation levels of the target word, prediction mismatches between bottom-up input and top-down predictions or the extent to which perceived input does not match with the current resonance state of semantic memory (see Lau et al., 2008; Kutas and Federmeier, 2011; Lotze et al., 2011; Rabovsky and McRae, 2014; Bornkessel-Schlesewsky and Schlesewsky, 2019). Thus, leaving aside the heterogeneous implementations of the proposed N400 models, an assumption common to all these accounts of the N400 is that its amplitude reflects the relative efficiency in processing stimulus or word properties in relation to the preceding context.

Although the majority of N400 studies report that reductions of N400 amplitude converge with reduced RT and error rates (or vice versa), there is also a non-negligible number of studies reporting diverging effects of N400 amplitude and behavioral measures. Many of the latter studies have investigated the processing of words either pre-activated/predicted *via* (lexical-)semantic priming, contextual predictability or a combination of both. The specific kind of divergence differs across studies, depending on whether: (i) N400 and behavioral measures show incongruent effect directions across measures or incongruent effect sizes, particularly nil effects in one vs. the other measure (e.g., Holcomb and Kounios, 1990; Kounios and Holcomb, 1992; Holcomb, 1993; Chwilla et al., 2000; Kiefer, 2001; Rolke et al., 2001; Federmeier et al., 2010; Debruille et al., 2013; differences with eye movements: Dimigen et al., 2011; Kretzschmar et al., 2015; Degno et al., 2019); or (ii) behavioral effects have reflexes in a biphasic pattern of N400 and (partly) overlapping positivity (e.g., Roehm et al., 2007; Bakker et al., 2015; Meade and Coch, 2017). For instance, in a study on lexical and semantic-priming effects on the processing of newly-learned vs. existing words, Bakker et al. (2015) found diverging effects of lexicality and semantic relatedness in response accuracy and ERPs elicited by target words in a word-list presentation. Specifically, the interaction between lexicality and semantic relatedness affected response accuracy such that error rates were higher for novel words related to their prime than unrelated ones, but not for existing words. RT, by contrast, showed only a main effect of semantic relatedness such that related targets were responded to faster, regardless of the type of input (novel word vs. existing word). The interaction between lexicality and semantic relatedness affected ERPs somewhat differently in that the N400 was sensitive to semantic relatedness only with existing words, exhibiting the typical amplitude reduction for related words. The posterior late positivity showed an enhanced amplitude for existing and novel words following related primes, although this was qualified by the time that had elapsed between the learning and the test session. Specifically, the posterior priming effect based on semantic relatedness was only found with novel words that could consolidate in long-term memory, while there was no difference with more recently acquired novel words. Thus, online processing effects reflected in the N400 did not show up in behavior, while the late positivity showed an interaction only partly compatible with RT. While the correlation of behavioral and ERP data was not central to the research reported in Bakker et al. (2015), the authors suggested that component overlap of N400 and the late positivity may account for the lack of a priming effect for novel words in the N400 time window.

Indeed, component overlap seems to be a plausible explanation given an earlier finding that, with increasing strength of semantic relatedness and contextual predictability, ERPs in the N400 time window become more positive, resulting in clearly visible P300 peaks for strongly related targets that can be actively predicted. This pattern was first reported in Kutas and Hillyard (1980) who showed that when context information and semantic relatedness converge to allow only one or a few candidates to felicitously end a sentence, N400 amplitude reduction seems to be overlaid with a P300. In other words, with high contextual constraint and a cloze probability of (nearly) 1 for the target, electrophysiological data are equivocal as to the ERP component driving amplitude modulations in the N400/P300 time window.

This pattern has been confirmed in a handful of ERP studies using the antonym paradigm (Bentin, 1987; Kutas and Iragui, 1998; Roehm et al., 2007; Federmeier et al., 2010) that provides strong semantic relatedness as well as high contextual predictability. Because antonyms are the logical endpoints on an opposition scale, antonym word pairs strongly prime each other. This effect can be strengthened with a sentence context such as *x is the opposite of y* or by using an experimental task that requires participants to think of or judge the antonymy relation between words, thereby increasing target cloze probability to nearly 1 (see Bentin, 1987; Roehm et al., 2007). Thus, from among the range of possible cloze probability values that a predictable target can have, the antonym paradigm picks up those with near-perfect cloze probability, yielding an almost binary distribution for predictable vs. unpredictable targets. Strikingly, even though this design revealed distinct P300 effects for expected antonyms and N400 amplitude increases for unpredicted non-antonyms across studies, the behavioral patterns do not converge with the ERPs. While some found that RT and error rates show facilitative effects for antonyms (Bentin, 1987), others found that non-antonym conditions fare better than antonyms behaviorally (Roehm et al., 2007; Federmeier et al., 2010). The lack of the typical behavioral priming effect for antonyms (i.e., reduced RT or error rates, see Neely, 1991) in some experiments is especially striking given that the ERP pattern is rather stable across studies.

This latter dissociation of P300 and behavioral measures in the antonym paradigm is also intriguing insofar as the P300/P3b, a domain-general positive-going potential that peaks about 250–500 ms after target onset and exhibits a posterior maximum (Polich, 2007), has been found to be sensitive to stimulus categorization and predictability and to show positive correlations with behavior. In particular, the P300 is elicited by motivationally significant target stimuli, especially those relevant for task performance (see reviews in Johnson, 1986; Nieuwenhuis et al., 2005, 2011; Polich, 2007). It has been linked to evidence accumulation for categorization, that is its amplitude is enhanced the more evidence from stimulus properties has been accumulated in order to make a decision on the stimulus category (O’Connell et al., 2012; Kelly and O’Connell, 2015; Twomey et al., 2015). As such it shows correlations with both stimulus-locked and response-locked brain activity (Verleger et al., 2005). More specifically, several studies have reported positive correlations between P300 latency and RTs (see review in Nieuwenhuis et al., 2005; for an example from language processing, see Sassenhagen and Bornkessel-Schlesewsky, 2015), as long as participants are instructed to emphasize response accuracy over speed (Kutas et al., 1977; but see Pfefferbaum et al., 1983).

In language processing, P300 latency varies with the absence or presence of a prediction match, especially when the target word is crucial to perform a categorization task with a binary choice (e.g., acceptability, sentence verification). For example, the P300 peaks earlier for the detection of a preferred (i.e., predicted) constellation than for a dispreferred or unpredicted one at various linguistic levels (see Haupt et al., 2008; Kretzschmar, 2010; Bornkessel-Schlesewsky et al., 2015; Graf et al., 2017). For instance, Graf et al. (2017) found that for grammatically correct vs. incorrect auxiliary choice in German sentences, P300 and acceptability judgments converged with grammatical auxiliary selection showing earlier P300 and higher acceptability ratings compared to ungrammatical selection. Similarly, in Roehm et al.’s (2007) study mentioned above, the P300 in response to predicted antonyms—the single possible sentence completion—peaked earlier than the P300 to unpredictable non-antonyms. Yet, when relevant stimulus properties conflict with one another and there is thus lower decision certainty during categorization, P300 amplitude is diminished. This is evidenced by some of the abovementioned studies investigating semantic relatedness. For instance, the P300 to non-antonyms in Roehm et al.’s (2007) study has a smaller amplitude when the non-antonym is semantically related to the predicted antonym compared to when it is unrelated (see “Experiment 1: Antonym Processing and ERPs” section below). Akin to what Bakker et al. (2015) reported for semantic priming for novel word meanings with a short consolidation time, P300 amplitude decreased for semantically related target words in Roehm et al.’s (2007) study. Importantly, however, behavioral data failed to converge with the ERP pattern, as antonyms did not show faster RT or higher accuracy than the other conditions.

In summary, both the N400 and the P300 appear to be sensitive to predictability during linguistic categorization: N400 amplitude and P300 latency each signal the presence or absence of a prediction match during target categorization, while semantic relatedness reduces the amplitude of both ERPs. Importantly, this pattern converges with proposals that the N400 indexes the processing of stimulus properties relevant for categorization (Bornkessel-Schlesewsky and Schlesewsky, 2019, including linguistic fit), while the P300 indexes the dynamics of the categorization process itself (Twomey et al., 2015). Hence, N400 and P300-related processed depend on the same input, but reflect partly independent cognitive operations. A cognitive interpretation in terms of processing efficiency, however, is elusive as behavioral patterns (facilitation vs. inhibition) diverge.

Now, while aligning ERP patterns with behavioral patterns descriptively *via* inspection of their respective effect directions and sizes is not uncommon, it clearly suffers from two methodological challenges, summarized in (i) and (ii) below:

(i) RT and accuracy are often measured with a single button press with substantial delay, i.e., seconds after the critical target engendering the ERP effect of interest. Standard RT measures thereby lack time-sensitive information about the development of the behavioral response or processing dynamics and reflect the unweighted sum of several online processes. Inferences associating behavioral data to brain activity are thus difficult to draw. Related to this, standard RT measures conflate the likelihood of retrieving the correct information from memory with the likelihood to retrieve some representation faster than others (see McElree, 2006). Specifically, participants may trade speed for accuracy (i.e., give faster responses with a higher ER) or vice versa. Thus, any comparison between ERPs and behavior is complicated by the unidimensional nature of standard RT measures. This seems especially disadvantageous in cases as described above, where two distinct ERP components may index the categorization of stimulus properties and it’s associated time-course.
(ii) N400 and P300 overlap in time and scalp topography. Thus, effects ascribed to either of the two components may also stem from processes related to the respective other component. That is, amplitude modulations in a given component under study may be the result of offsets introduced by an adjacent component (additive component overlap), reflective of modulations within a given component or a mixture of the two (multiplicative component overlap). This may interfere with the standard statistical analysis of ERPs, in which the two components are often investigated with voltage information from one and the same time window. From this perspective, where two components collapse towards a unidimensional voltage measure, inferences from electrophysiological to behavioral data are difficult to draw.

For the first issue, we propose that the SAT paradigm is better suited than standard RT measures to discover the time-course of decision-making during sentence categorization. The SAT method measures participants’ binary decisions at varying latencies after the onset of the critical stimulus, thus capturing the development of categorization when information consolidates over time. In addition, with the SAT paradigm, categorization speed and accuracy can be dissociated analytically, as decision-making is reflected in three independent response parameters: asymptote, rate and intercept (Wickelgren, 1977). Response accuracy (measured in *d’* units) is reflected in the asymptote parameter. Speed parameters indicate when participants depart from chance level (intercept) and how quickly they achieve asymptotic performance (rate), i.e., their final decision state. The SAT paradigm may, therefore, allow for a more fine-grained comparison of ERPs and behavioral measures of processing efficiency because both data types capture some dimension of processing dynamics.

The second issue is more difficult to address in the presence of a biphasic ERP pattern. However, by applying modern statistical methods one can investigate the independence of the N400 and P300 signals. In using the antonym paradigm, the strong theory-based prediction of a P300 for a single possible completion and an N400 for violations of that prediction as well as the use of single-trial analyses incorporating both subject and item variation excludes the possibility that this biphasic pattern is artifactual (see Tanner et al., 2015 for filter artifacts, Tanner and Van Hell, 2014 for misleading grand averages in the case of interindividual differences). Joint modeling of both components in the biphasic response, either through careful selection of covariates or through multivariate models, allows for adjusting for the influence of each component and modeling their covariance, respectively. For introducing our novel modeling approach and keeping model complexity reasonable, we focus on temporal overlap of the N400 and P300 occurring in the largely overlapping time windows (approximately 250–500 ms post target onset), as the ERP methodology in sentence and word processing is still more often used to make inferences based on the temporal dimension (i.e., when information is processed in the brain), rather than on an integrated spatiotemporal profile (but see Nieuwland et al., 2019). Hence, we will both disregard topographic overlap between the two components (although we note that this may be a useful extension of the approach) and the late positivity following the N400 for disconfirmed predictions, as, currently, it is not settled whether this is one component or several depending on topographical distribution (see Van Petten and Luka, 2012; Leckey and Federmeier, 2019).

We collected ERP and behavioral SAT data in two separate experiments to illustrate the feasibility of our proposal sketched above. Experiment 1 using ERPs serves as a replication of previous studies investigating categorization of predictable target words in sentences and Experiment 2 is complementary to standard RT measures accompanying ERP recordings. In both experiments, we used the antonym paradigm as presented in Roehm et al. (2007) and asked participants to judge sentences for acceptability on a binary (yes/no) scale.

## Experiment 1: Antonym Processing and ERPs

Experiment 1 serves as a replication of the first experiment reported in Roehm et al. (2007). Roehm et al. (2007) investigated the comprehension of antonym pairs in a strongly constraining sentence “x is the opposite of y,” with *x* being the prime and *y* the target antonym (see example 1), where participants were asked to verify the antonymy relation between prime and target. The prime-target word pair is related *via* an antonymy relation and target predictability additionally strengthened *via* the sentence fragment occurring in between the two antonyms. The antonym pairs (example 1a) were contrasted with two types of violation, semantically related non-antonym targets (example 1b) and semantically unrelated non-antonyms (example 1c). This paradigm essentially contrasts the two variables predictability and semantic relatedness. In terms of predictability, only the antonym target is predictable from context, whereas both non-antonym endings are equally unexpected (see Roehm et al., 2007 for details about stimuli norming). Regarding semantic relatedness, related non-antonyms belong to the same semantic field or category as the expected antonym, whereas unrelated non-antonyms do not (see Löbner, 2013). Hence, semantic relatedness can be equated with semantic priming *via* an automatic spread of activation in long-term memory (see Collins and Loftus, 1975), while sentence contexts pushe predictions about what word can plausibly and truthfully end the sentence.

(1) Example sentences of the antonym paradigm employed in Experiment 1 by Roehm et al. (2007; target words are underlined)

a. Black is the opposite of white.
b. Black is the opposite of yellow.
c. Black is the opposite of nice.

Roehm et al. (2007) found that strongly predicted antonyms, such as *white* in example (1a), engendered a P300 between 240 and 440 ms after target onset, which overlapped with the N400 that showed increased amplitudes for the two non-antonym conditions. The N400 effect was less pronounced for related non-antonyms from the same semantic category as the antonym (example 1b) vs. unrelated ones (example 1c). Additionally, N400 effects to non-antonyms were followed by a late positivity, which was stronger for unrelated non-antonyms than related non-antonyms at posterior electrode sites. These ERP effects are summarized in the top two rows of Table 1.

**Table 1:** Summary of significant differences in Experiment 1 by Roehm et al. (2007).

ERP components and time windows analyzed	Effects
N400/P300 time window (240–440 ms)	antonym (P300) < related (N400) < unrelated (N400)
Late positivity time window (500–750 ms)	global distribution: antonym < related
	antonym < unrelated
	posterior distribution:
	related < unrelated
Error rate	unrelated < antonym < related
Response time	unrelated < antonym < related

*Note: “a < b” means a significantly less negative amplitude (hence an increased P300 or reduced N400), lower error rate or shorter response time for a vs. b*.

Although the antonym paradigm as described above includes a binary contrast between perfectly predictable targets and unpredictable violations, the ERP findings largely converge with previous studies which also manipulated target predictability and semantic relatedness. P300 responses to strongly predictable target words with near-perfect cloze probability (i.e., single possible completions, which is also the case for antonyms in sentence context), have been reported for word-list and sentence processing in English (Kutas and Hillyard, 1980; Bentin, 1987; Kutas and Iragui, 1998; Federmeier et al., 2010). Data from studies employing a broader range of cloze probability scores further support the pattern obtained in Roehm et al.’s (2007) experiment. P300 amplitude reductions as a consequence of semantic relatedness between target and prime have been previously found in a word-list experiment (Bakker et al., 2015). N400 amplitude increases to prediction violations and amplitude reductions due to semantic relatedness or category membership were reported for unexpected or unprimed words other than antonyms (e.g., Federmeier and Kutas, 1999; Bakker et al., 2015; Meade and Coch, 2017).

Overall, this pattern of results support the above considerations of how semantic relatedness/priming and predictability distinguish the three critical conditions in Roehm et al.’s (2007) design, and of how N400 and P300 ERPs may index different aspects of linguistic categorization. The P300 indexes stimulus categorization and emerges within the N400 time window for prediction matches, especially when predictability and semantic relatedness converge to single out the expected target, here the second antonym word. In the case of prediction mismatches, P300 peak latency follows the N400 and its amplitude is reduced when semantic relatedness interferes with categorizing the stimulus as an unexpected non-antonym. The N400, in turn, overlays the P300 component when unpredicted stimulus features need to be processed. It shows facilitative effects of semantic relatedness for prediction mismatches, as priming facilitates the processing of stimulus features due to spreading activation and this is independent of the ensuing categorization.

Yet, the behavioral data from the antonymy verification task in Roehm et al.’s (2007) first experiment showed a pattern that is difficult to integrate with the above functional description of the ERP data, especially regarding the P300. For both ER and RT, unrelated violations (example 1c) were judged fastest and most accurate, whereas related violations (example 1b) were slowest and most error-prone. Antonyms fell in between the two prediction violations. Hence, behavioral data do not show clear evidence for a behavioral advantage of predictability that would mirror the P300 to antonyms, whereas they indicate that semantic relatedness of unpredicted non-antonyms has a negative effect, similar to the amplitude reduction of the late P300 in response to related non-antonyms. Conversely, the data are not suggestive of a facilitative behavioral effect of semantic relatedness that would mirror the N400 effect.

Given that the current experiment is a rather direct replication attempt of Roehm et al.’s (2007) first experiment, we expect to replicate both the ERP and behavioral data patterns.

### Methods

#### Participants

Twenty participants (14 females, mean age: 23.15 years, SD: 2.60) from the University of Cologne participated for payment (8€/hour) or course credit. All participants were monolingual native speakers of German and reported normal or corrected-to-normal vision and no history of psychological or neurological disorders. All were right-handed as assessed with an abridged German version of the Edinburgh handedness test (Oldfield, 1971). The protocol for ERP experiments conducted in the lab is approved by the Ethics Committee of the German Society of Linguistics (DGfS; #2016-09-160914). Participants gave written informed consent prior to their participation.

#### Materials

We used the same sentence stimuli as in Roehm et al. (2007) and made publicly available in Roehm (2004).

#### Apparatus and Procedure

EEG was recorded from 55 Ag/AgCl electrodes (ground: AFz; 10-10 system) fixed at the scalp by means of an elastic cap (Easycap GmbH, Herrsching, Germany). EOG was recorded from three additional pairs of electrodes placed at the outer canthus, supraorbital and infraorbital of each eye. The sampling rate was 500 Hz (BrainAmp DC, Brain Products, Gilching, Germany). Data were referenced to the left mastoid for recording. Impedances were kept below 5 kOhm.

Before the experiment, participants were instructed to judge in an acceptability task whether the sentence is correct or not, and were given 10 practice trials to familiarize with the task. Note that we did not use the kind of antonym verification judgment employed in the original study, as this was less optimal for Experiment 2 (see “Apparatus and Procedure” section below). Participants were seated in a sound-attenuated booth, at a distance of approximately 100 cm from a 24-inch monitor. Sentences were displayed centered on the screen and in black font (Verdana, 28 pt) against a light-gray background. Rapid serial visual presentation (RSVP) closely followed the specifications given for Roehm et al.’s (2007) first experiment [with the exception of the inter-trial interval (ITI)]. Each trial began with the presentation of a fixation star, presented for 2,000 ms, to focus participants’ attention to the upcoming sentence. Sentences were then presented word by word, with 350 ms per word and 200 ms interstimulus interval (ISI). After the sentence-final target word, a blank screen was presented for 650 ms and then replaced with question marks indicating that participants could now give their judgment with one of two buttons on a game pad. Maximum response time was 3,000 ms. The ITI was 2,000 ms (vs. 2,250 ms in the original study). Assignment of response buttons (correct vs. incorrect) to the right and left hand was counterbalanced across participants.

Items were presented in four lists, each containing 80 sets of antonym sentences and 40 sets in each of the two non-antonym conditions. Participants were randomly assigned to one of the lists, which were presented in one of two pseudorandomized orders.

#### Analysis and Results

EEG data were processed with MNE-Python 0.17.1 (Gramfort et al., 2013). Data were re-referenced to linked mastoids offline and bandpass filtered from 0.1 to 30 Hz (bandpass edge, hamming-windowed FIR, with zero-phase achieved *via* compensation for the group delay). Bipolar horizontal and vertical EOG were computed, and the very most anterior (AF*x*), posterior (P*x*) and temporal electrodes (TP*x*) data were excluded from further analysis. The continuous EEG was then divided into epochs extending from 200 ms before onset of the critical word until 1,200 ms after onset. Trials where the peak-to-peak voltage difference exceeded 150 μV in the EEG or 250 μV in the bipolar EOG were excluded from further analysis. Additionally, flat-line trials (where the peak-to-peak voltage in the EEG was less than 5 μV) and trials where the absolute voltage exceeded 75 μV were excluded. No baseline correction was performed as part of the preprocessing. However, the trial-wise mean voltage pre-stimulus interval (−200 to 0 ms) was used to baseline correct for plotting purposes and entered as a covariate into the statistical analyses (see Alday, 2017). The preprocessed EEG data along with analysis source code is available on the Open Science Framework (OSF; see “Data Availability Statement” below).

Subsequently, trials with an incorrect or timed-out behavioral response were also excluded (2%–5% of trials on average per condition). As this reflects ceiling performance, we did not further analyze behavioral data from the EEG experiment. However, numerical values for both RT and accuracy rates are highly similar to the original data, as shown by grand means and standard errors: highest accuracy rates (0.98 ± 0.012) were obtained for the unrelated non-antonyms, followed by the antonym condition (0.95 ± 0.016). Related non-antonyms were judged with lowest accuracy (0.94 ± 0.012). RT to correctly answered trials confirmed this pattern, with fastest RT (in milliseconds) for unrelated non-antonyms (450 ± 37), slowest RT for related ones (550 ± 57), and antonyms falling in between the two (470 ± 31).

In total, 2,898 trials across 20 subjects remained for an average of 145 trials per participant (72 antonym, 36 related, 37 unrelated).

**Figure 1:**
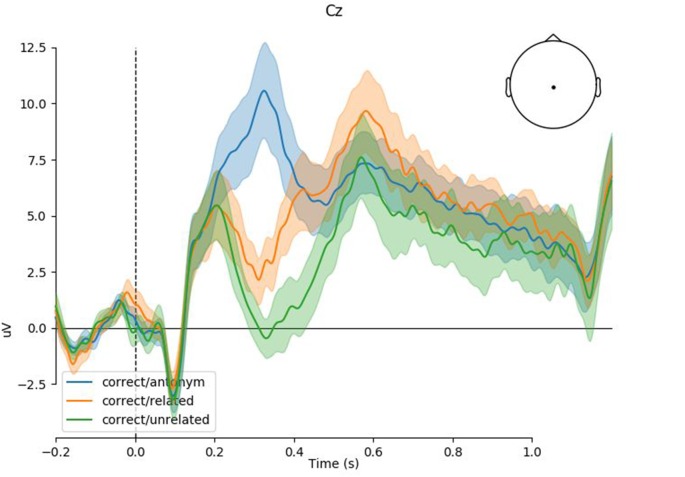
Event-related potential (ERP) time-course at Cz (Experiment 1). Shaded regions indicate 83% confidence intervals of the grand mean; non overlap is equivalent to significance at the 5% level. Positivity is plotted upwards.

**Figure 2:**
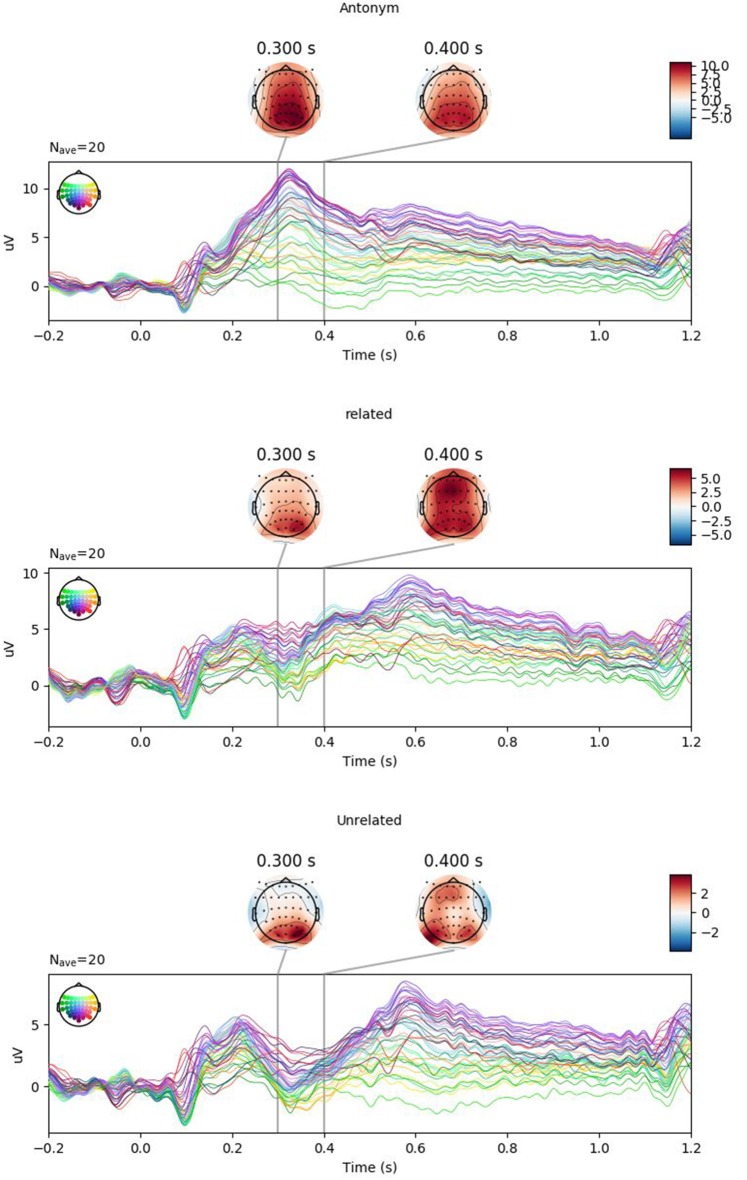
Topographies of ERPs by condition (Experiment 1). Positivity is plotted upwards. Note the clear positivity peaking at 300 ms in the antonym condition (top) as well as the negativity around 400 ms in the related (middle) and unrelated (bottom) conditions.

Figure 1 shows the grand-average response at Cz with 83% confidence intervals. Non-overlap of 83% confidence intervals corresponds to significance at the 5% level, or equivalently, the 95% confidence interval of the difference not crossing 0. As expected and observed in previous studies, we see a clear P300 for the antonym condition and a graded N400 for the related and unrelated violation conditions. As shown in the by-condition plots (Figure 2), the topographies of these components correspond to the typical centro-parietal characterization of the P300 and N400 components.

As the purpose of this study was not to examine the topography of well-characterized components, we restrict ourselves for simplicity and computational efficiency in the cross-method analysis to a centro-parietal region of interest (ROI) comprising 26 electrodes (C1, C2, C3, C4, C5, C6, Cz, CP1, CP2, CP3, CP4, CP5, CP6, CPz, P1, P2, P3, P4, P5, P6, P8, Pz, PO3, PO4, POz, Oz) that were least affected by artifacts across participants and trials, and that typically show maximum activity for the visually-evoked N400 effect (e.g., Johnson and Hamm, 2000) and P300 effect (e.g., Verleger et al., 2005), respectively. We used single trial mean voltage for the *a priori* chosen P300 (200–300 ms post-stimulus, as this more adequately captured P300 activity for antonyms, see Bentin, 1987; Roehm et al., 2007) and N400 (300–500 ms post-stimulus, see Kutas and Federmeier, 2000), and this was used as EEG measure in all analyses below. While the choice of component time windows reduces overlap, it does not eliminate it, if for no other reason than a larger P300 serves as an offset for a subsequent N400 component.

We analyzed these single-trial data with linear mixed-effects models using lme4 (v1.1-20, Bates et al., 2015b), with fixed effects for the mean voltage in the baseline window (see above; Alday, 2017) and condition as well as their interaction. All EEG measures were transformed to the standard deviation scale, and condition was sequential difference coded such that the contrasts related > antonym and unrelated > related are directly represented in the coefficients.

Random effects consisted of by-item intercepts and by-subject intercepts and slopes for condition. This models random variation in the lexical material as well as between-subject differences in the overall and by-condition EEG response. While this random-effect structure is not maximal in the sense of Barr et al. (2013), the data do not support a more complex structure and we do not expect additional variation along the omitted dimensions (see Bates et al., 2015a; Matuschek et al., 2017). Moreover, for the present study, where model comparison is more important than significance, any potential issues with anti-conservative significance of fixed-effects component are irrelevant.

Statistical analysis confirms the visual impressions that the present data replicate the findings of Roehm et al. (2007; see Tables s 2, 3). In particular, we observe a graded response in both the N400 and P300 time windows, with the main effect for condition reflecting a significant difference between related and unrelated (the reference level) as well as antonym and unrelated.

**Table 2:** Linear mixed effect model for the P300 time window (Experiment 1).

**AIC**	**BIC**	**logLik**	**deviance**	**df.resid**
7,743	7,827	−3,858	7,715	2,884
**Scaled residuals**:				
**Min**	**1Q**	**Median**	**3Q**	**Max**
−4.45	−0.64	−0.02	0.64	3.61
**Random effects**:				
**Groups**	**Term**	**Std.Dev.**	**Corr**
item	(Intercept)	0.11		
subj	(Intercept)	0.25		
	related >	0.45	−0.16	
	antonym
	unrelated >	0.10	−0.98	0.34
	related
Residual		0.89		
Number of obs: 2,898, groups: item, 80; subj, 20.				
**Fixed effects**:
	**Estimate**	**Std. Error**	***t*-value**	
(Intercept)	−0.089	0.059	−1.5	
baseline	0.09	0.02	4.6	
related >	−0.47	0.11	−4.3	
antonym
unrelated >	0.044	0.052	0.84	
related
baseline:related >	0.11	0.045	2.4	
antonym
baseline:unrelated >	−0.022	0.05	−0.45	
related				

*The response is the trial-wise mean amplitude at a centro-parietal ROI in the time window 200–300 ms, the baseline is the trial-wise mean amplitude in the pre-stimulus window −200 to 0 ms. EEG measures are centered and scaled. Model estimated using maximum likelihood (i.e., REML = FALSE) and the bobyqa optimizer*.

**Table 3:** Linear mixed effect model for the N400 time window (Experiment 1).

**AIC**	**BIC**	**logLik**	**deviance**	**df.resid**
7,002	7,086	−3,487	6,974	2,884
**Scaled residuals**:			
**Min**	**1Q**	**Median**	**3Q**	**Max**
−3.29	−0.66	0.03	0.65	5.06
**Random effects:**			
**Groups**	**Term**	**Std.Dev**.	**Corr**	
item	(Intercept)	0.14		
subj	(Intercept)	0.30		
	related >	0.37	−0.41	
	antonym
	unrelated >	0.12	0.40	0.68
	related
Residual		0.78		
Number of obs: 2,898, groups: item, 80; subj, 20.				
**Fixed effects**:
	**Estimate**	**Std. Error**	***t*-value**	
(Intercept)	−0.14	0.071	−1.9	
baseline	−0.17	0.017	−9.7	
related >	−0.55	0.091	−6	
antonym
unrelated >	−0.32	0.05	−6.4	
related
baseline:related >	0.064	0.039	1.7	
antonym
baseline:unrelated >	−0.0053	0.044	−0.12	
related				

*The response is the trial-wise mean amplitude at a centro-parietal ROI in the time window 300–500 ms, the baseline is the trial-wise mean amplitude in the pre-stimulus window −200 to 0 ms. EEG measures are centered and scaled. Model estimated using maximum likelihood (i.e., REML = FALSE) and the bobyqa optimizer*.

### Discussion of Experiment 1

The current experiment aimed at replicating the findings from Experiment 1 in Roehm et al. (2007). In line with the original study, we find that the conditions elicit distinct ERP responses depending on target predictability and semantic relatedness. Between 200 and 300 ms post target onset, antonyms (*white*) engender a pronounced P300, while related non-antonyms (*yellow*) and unrelated non-antonyms (*nice*) both elicit an N400 effect between 300 and 500 ms post target onset. The N400 for unrelated non-antonyms was larger than the one for related non-antonyms. In addition, visual inspection suggested that the N400 in the two non-antonym conditions was followed by a late positivity, which was, however, less pronounced than the early P300 for antonyms.

## Experiment 2: Antonym Processing in The Speed-Accuracy Trade-Off Paradigm

As discussed above, with standard behavioral measures of response time and accuracy, data interpretation can be complicated by the fact that response time and accuracy may vary in their relationship across participants and on a trial-to-trial basis. That is, participants may trade response speed for accuracy or vice versa, for instance when adapting their decision criterion to the experimental task at hand (see Kutas et al., 1977; Wickelgren, 1977).

In Experiment 2, we used the SAT paradigm (Wickelgren, 1977) that measures participant’ response accuracy as a function of their response speed and that has been successfully employed in a number of previous investigations on various phenome in sentence processing (e.g., McElree et al., 2003; Bornkessel et al., 2004; Martin and McElree, 2009; Bott et al., 2012). We adopted the SAT paradigm as it allows independent estimates of processing accuracy and dynamics. Participants give speeded binary acceptability judgments in response to short signal tones, presented at varying latencies from critical word onset. Individual *d’* scores are computed as a measure of sensitivity to stimulus properties and the development of response accuracy depending on time is described with three SAT parameters. Asymptote (λ) reflects the highest level of participants’ accuracy. Response speed is reflected in two parameters: the intercept (δ) is the point when participants depart from chance level in giving accurate responses and the rate (β) reflects the speed with which they reach their individual asymptotic performance. Thus, the categorization process can be described with multidimensional behavioral data (contrasting with standard RT measures).

We predict that the three conditions in the antonym paradigm should exhibit distinct SAT profiles. Recall that only antonym pairs are predictable, whereas the two non-antonym conditions are unpredictable from the preceding context. Related non-antonyms are distinct from unrelated ones by being semantically related to the correct and predicted antonym. Specifically, there are two possible general predictions based on whether: (a) predictability dominantly determines categorization or (b) whether predictability and semantic relatedness interactively determine decision. If only predictability matters for categorization, then decisions for antonyms should be more accurate and faster than the other two unexpected sentence endings. If, however, in addition to predictability semantic relatedness is taken into account for categorization, we expect a slightly different pattern. Specifically, semantic relatedness may be helpful in stimulus processing under the premise of spreading activation of the expected antonym to other category members (see Collins and Loftus, 1975; Kretzschmar et al., 2009). However, from the perspective of categorization, relatedness may likewise be conceived of as an intervening factor in deciding on whether, e.g., *yellow* is or is not an antonym to *white*. By definition, category members share semantic features which makes their categorization less easy for related non-antonyms as they are less distinct from the expected antonym by means of shared features. Features shared between the expected target and a competitor (cue overload) has been shown to make other categorization at the sentence level (e.g., subject-verb agreement) harder, leading to lower accuracy and slower processing dynamics in the SAT curve (McElree et al., 2003; Johns et al., 2015). Thus, if semantic relatedness is indeed an intervening factor in categorization, related non-antonyms should show lower asymptote and slower processing dynamics compared to the other two conditions because it is more difficult to achieve a stable decision point. Antonyms and unrelated non-antonyms should reveal identical patterns from this perspective because decision can be reliably made due to a prediction match (i.e., identical feature set of expected target and perceived target) or a mismatch with unshared features (i.e., maximally distinct feature set for unrelated non-antonyms compared with the expected antonym).

Note that our predictions for differences in processing speed are somewhat speculative because previous results on the retrieval of semantic cues in sentence processing using the SAT method have provided mixed findings on differences in processing dynamics (e.g., McElree et al., 2003; Martin and McElree, 2009; Johns et al., 2015).

### Methods

#### Participants

Sixteen participants (nine females, mean age: 24.44 years, SD: 2.61) from the Universities of Marburg and Mainz participated in Experiment 2. Participants were paid 7€/hour for their participation. None of them participated in Experiment 1. All participants were native speakers of German (15 monolingual, one bilingual) and reported normal or corrected-to-normal vision and no history of psychological or neurological disorders. Experiment 2 was not accompanied by an ethics vote but was conducted in line with national and institutional guidelines, as specified by the rules of the German Research Foundation (DFG). Specifically, behavioral non-invasive experiments with healthy young adults (between 18 and 65 years) do not require one as long as they pose no risk or physical/emotional burden to participants and as long as participants are debriefed after participation. See “Ethics Statement” for details. Participants gave written informed consent prior to their participation. One participant was excluded from analysis because of below-chance performance in response accuracy.

#### Materials

We selected 20 sets of items from the original 80 sets used in Experiment 1. The number of items was reduced in order to keep the number and length of experimental sessions at a reasonable size, as SAT experiments are typically conducted with many more filler items than ERP experiments. There were eight items with adjectival pairs and six items with verbal and nominal pairs each. The order of prime and target words was reversed to meet methodical requirements of the SAT procedure: in order to obtain a useful estimate of processing speed, the critical target word needs to be lexically identical across conditions. By reversing prime and target words in the original item sets, we could achieve that (see example 2). Each item occurred in one of the three critical conditions (antonym, related and unrelated non-antonyms) and in a fourth repetition condition that was used for *d’* scaling.

(2) Example set of items in Experiment 2

a. antonym condition: *Klein ist das Gegenteil von groß*. “Small is the opposite of big.”
b. related non-antonym: *Dick ist das Gegenteil von groß*. “Thick is the opposite of big.”
c. unrelated non-antonym: *Grün ist das Gegenteil von groß*. “Green is the opposite of big.”
d. repetition: *Groß ist das Gegenteil von groß*. “Big is the opposite of big.”

With the acceptability task used here, the antonym condition is the only one requiring an “acceptable” (yes) response. There were 40 filler items with a comparable sentence beginning (“x is the y”) to reduce the saliency of the frame “x is the opposite of y”; 20 of them contained semantic or syntactic (gender, category) violations at various positions in the sentence, thus requiring an “unacceptable” (no) response. There were further 336 filler sentences of varying structures from other experiments, 184 of which required an “unacceptable” (no) response. From the total of 464 sentences, 264 (57%) required an “unacceptable” (no) response, 200 (43%) an “acceptable” (yes) response^1^. Items in the four critical conditions constituted 17% of all trials.

#### Apparatus and Procedure

Items were presented in black font (Monaco, size: 38 pt) on a white background, centered at the screen of a 21-inch monitor. Participants were instructed to read the sentences and to judge them for acceptability (yes/no) upon hearing a response signal. We did not use an antonym verification task as in the original study by Roehm et al. (2007) because this would have not worked for the various filler items.

We employed the multiple response-SAT paradigm (see Bornkessel et al., 2004; Martin and McElree, 2009). Fifteen response tones (2,000 Hz, 50 ms duration) followed each sentence, with the first two tones preceding the onset of the target word that provides the essential piece of information to judge acceptability. Participants had to give their response within 300 ms following each tone. Each trial began with a fixation star presented for 400 ms and an ISI of 1,000 ms. Next, participants saw which of the two response buttons (y and n on the keyboard) would serve as the default button for the responses in which they could not yet give a certain answer (see Bornkessel et al., 2004). Occurrence of the default buttons was equibalanced within and across conditions. Then, sentences were presented word-by-word at a fixed presentation rate of 300 ms/word and with an ISI of 100 ms. Before the onset of the sentence-final target word, the first two response tones were presented right after the offset of the pre-final word, and participants had to press the default button as a response within 300 ms following each of the two tones. Participants were instructed to switch to the y button for “acceptable” responses or the n button for “unacceptable” responses as soon as they could make a decision after seeing the target word on screen. The next trial began after an ITI of 1,500 ms.

The items were allocated to two lists; each list (containing 232 trials) was presented in eight blocks with short breaks in between. The first session additionally comprised a practice with 50 sentences unrelated to the experimental items, in which participants were trained to respond within 300 ms after tone onset. Participants took part in the sessions on two consecutive days.

#### Analysis and Results

Before analysis, the data from all participants were preprocessed to remove invalid data points. Due to recording bugs in presentation, some trials contained excessively long pauses before or during tone presentation. These trials were excluded from analysis (3.3% of trials), as were timed-out responses that did not occur within 300 ms after signal tone offset (less than five responses per condition on average across participants and latencies). The preprocessed SAT data along with analysis source code is available on the OSF (see “Data Availability Statement” below).

**Figure 3:**
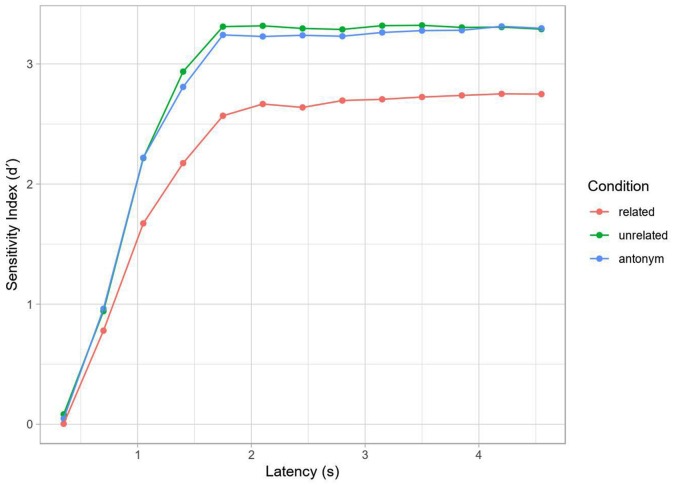
Speed-accuracy tradeoff (SAT) by condition (Experiment 2). Curve computed on grand average data. Note the lower asymptotic performance in the related condition, but otherwise similar dynamics.

For an initial assessment of behavioral performance, accuracy was computed for each decision point during the response interval (per participant and condition), using d’ as a sensitivity measure. Hits were defined as yes/“acceptable” responses to the antonym condition (example 2a) and no/“unacceptable” responses to the two non-antonym conditions (examples 2b, c). False alarms were defined as yes-responses to the repetition condition (example 2d). The resulting mean SAT curve is shown in Figure 3. In terms of percentage correct, the identity, unrelated and antonym conditions all reached ceiling (respective grand mean accuracies and standard errors at the final tone: 0.97 ± 0.027, 0.96 ± 0.022, 0.97 ± 0.008), while the related condition showed slightly worse but still high performance (0.83 ± 0.036), with the decreased performance perhaps reflecting interference and decision uncertainty (discussed more below).

**Figure 4:**
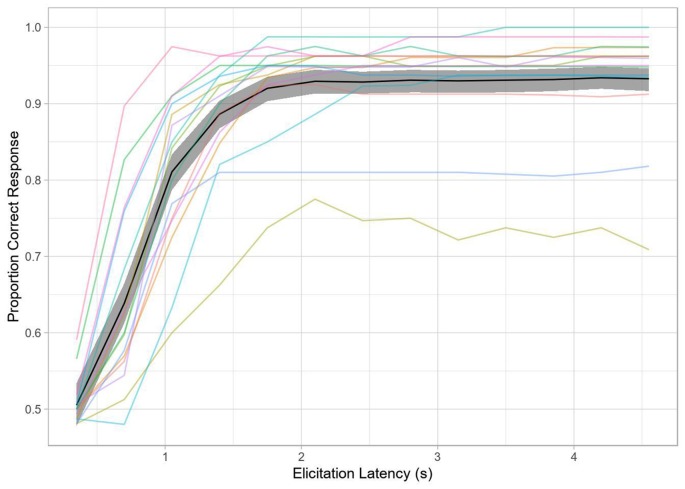
Accuracy over time (Experiment 2). The black line represents grand average accuracy across all conditions, with the shaded region indicating the 95% bootstrapped confidence interval of the grand mean. The colored lines indicate single-subject performance.

In contrast to traditional SAT analysis using within-subject curve-fitting to a subject’s d’ time-course with an exponential decay of error towards an asymptote, we used mixed-effects logistic regression with by-trial accuracy to model the SAT (see Davidson and Martin, 2013). This method has a couple of advantages for the present study: (1) we are not dependent on aggregation and can thus model item variance as well as trial-by-trial fluctuation in RT to each tone; and (2) we can model all subjects and their associated variance in a hierarchical fashion, allowing for partial pooling and shrinkage. This should yield more robust inferences. The overall time-courses for both methods are comparable, as seen in Figures 3, 4.

Fixed effects consisted of log-transformed total RT (tone latency + response time to that tone), condition and their interaction. Condition was sequential-difference coded in the same way as for the EEG data. Again, similar to the EEG data, random effects consisted of by-item intercepts and by-subject intercepts and slopes for log RT.

Although performance for later latencies was generally near ceiling, the related condition showed a significantly lower asymptotic performance than the other conditions (as shown in the combination of the intercept, and interaction effects for condition, Tables s 4, 5, see also Figure 3) and a slower ramp-up (as shown in the interaction effects for condition and log RT). This is comparable to a difference in the asymptote and rate parameters in traditional SAT analysis.

**Table 4:** Generalized linear mixed effect model for the speed-accuracy tradeoff (SAT) data (Experiment 2) based on condition.

**AIC**	**BIC**	**logLik**	**deviance**	**df.resid**	
7,293	7,367	−3,637	7,273	11,484	
**Scaled residuals**:
**Min**	**1Q**	**Median**	**3Q**	**Max**	
−15.05	0.09	0.2	0.37	2.95	
**Random effects**:
**Groups**	**Term**	**Std.Dev.**	**Corr**		
item	(Intercept)	0.65		
subj	(Intercept)	2.81			
	logRT	0.45	−0.996		
Number of obs: 11,494, groups: item, 20; subj, 15.					
**Fixed effects**:
	Estimate	Std. Error	*z*-value	Pr (>|z|)	
(Intercept)	−10	0.81	−12	1.3e-35	***
related > antonym	6.4	0.73	8.8	1.4e-18	***
unrelated > related	−5.8	0.73	−13	1.2e-15	***
logRT	1.7	0.13	13	1.6e-39	***
related > antonym:logRT	−1.1	0.1	−10	1.5e-25	***
unrelated > related:logRT	0.98	0.1	9.7	3.3e-22	***

*Dependent variable is the response accuracy, correspondingly the model family is binomial with a logit link. RT is the total reaction time, i.e., the response tone latency plus the reaction time to that tone. Model fit by maximum likelihood using the Laplace approximation and the bobyqa optimizer*.

**Table 5:** Comparison of slopes across conditions in the SAT model.

Contrast	Estimate	SE	*z*-value	*p*-value
antonym–related	1.0564	0.101	10.449	<0.001
antonym–unrelated	0.0801	0.116	0.688	0.7703
related–unrelated	−0.9763	0.101	−9.690	<0.001

*Marginal trends were computed using marginal means. The Tukey method was used to adjust p-values for three comparisons*.

### Discussion of Experiment 2

Experiment 2 is, to the best of our knowledge, the first experiment to investigate antonymy processing in the SAT paradigm. We hypothesized that speed and accuracy parameters are differentially influenced by the conditions, either due to predictability alone or due to an interaction of predictability and semantic relatedness. We found significant differences between conditions both in asymptotic performance and in processing dynamics (reflected in rate). Related non-antonyms were rated less accurately and at a slower rate than the other two conditions that did not differ from each other. The results thus suggest that predictability alone does not influence processing accuracy and speed in the antonym paradigm, because antonyms did not differ from both non-antonym conditions. Rather, semantic relatedness and predictability interacted such that relatedness made the evaluation of a target word as a prediction mismatch more difficult.

These findings support and refine previous behavioral data obtained in the antonym paradigm. The SAT data confirm that related non-antonyms are in fact more difficult to judge, as reflected both in RT and accuracy. This lends further support to our hypothesis that semantic relatedness interferes with categorization in that only related non-antonyms contain information that impede an unequivocal categorization. At the same time, the SAT data do not reveal significant differences between antonyms and unrelated non-antonyms as previously found with standard RT measures. This can be explained with the absence of semantic relatedness in the violation condition: unrelated non-antonyms are easily categorized as a mismatch because there is no overlap in semantic features with the expected antonyms. Hence, the SAT profile seems to be mainly determined by the ease of categorizing the perceived input as an antonym, rather than by the processing of (predictable or semantically related) linguistic properties *per se*.

Hence, one can conclude that, in the antonym paradigm, processing semantic relatedness—as revealed by reductions in N400 amplitude—does not influence behavioral signatures in a similar vein, i.e., it does not lead to faster or more accurate performance. Rather, semantic relatedness is an intervening factor for categorization, as we have suggested based on its negative effect on P300 amplitude (see “Experiment 1: Antonym Processing and ERPs” above). From this perspective, the SAT data seem more in line with the ERP data than standard RT measures.

Yet, with separate analyses we can still not directly relate the two data sets to each other. Therefore, we conducted a joint analysis of the SAT and EEG data to investigate whether behavioral performance was driven by N400-related processes, P300-related processes or both.

### Modeling SAT Dynamics as a Function of ERP Data

In addition to the direct modeling of the SAT response as a function of condition, we can also model the SAT response as a function of the mean ERP from the EEG experiment. For this model, fitted values by condition were extracted from mixed-effects models for the P300 and N400 and then aggregated to yield a single value for each component in each condition. These values are then used instead of the categorical predictor in an otherwise identical mixed-effect model for the SAT response. The difference in item sets (the EEG item set was larger) and participants, as well as the aggregation step, ensure that these values are not merely fitting within experiment item or participant variation, but rather capturing population-level dynamics.

**Figure 5:**
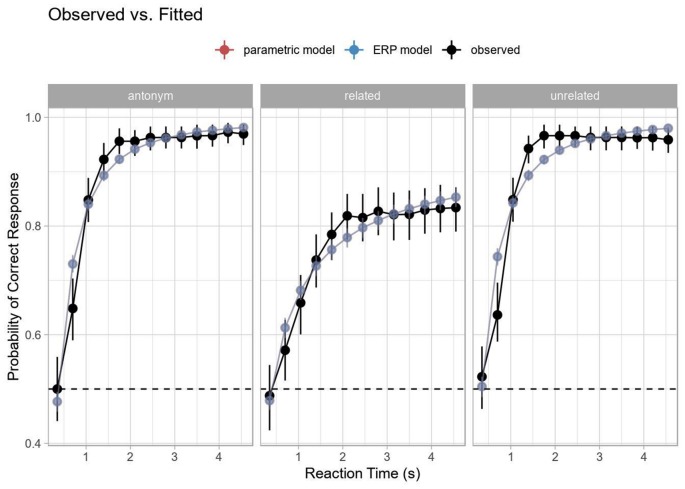
Comparison of model fit. The parametric and ERP-based models yield identical fits, shown here as perfect overlap (evident in the apparent color being a mixture of the blue and red of the individual colors in the legend), and fit the overall shape of the data well. Error bars indicated 95% bootstrap confidence intervals computed on the display (response) scale.

The resulting model (Table 6) is identical in fit to the model based on the categorical condition codes (see Figure 5 and the AIC and logLik values in Tables 4, 6). At first this may seem surprising, but this model has an identical number of parameters and differs in practice only in its design matrix that no longer codes condition directly but rather the electrophysiological “encoding” of (the response to) the condition. This decomposes the different processes present in each condition—much in the same way that independent components in ICA present the same data as the original channel-wise EEG yet reveal insights about latent structure.

**Table 6:** Generalized linear mixed effect model for the SAT data (Experiment 2) based on event-related potential (ERP) responses.

**AIC**	**BIC**	**logLik**	**deviance**	**df.resid**	
7,293	7,367	−3,637	7,273	11,484	
**Scaled residuals**:
**Min**	**1Q**	**Median**	**3Q**	**Max**	
−15.05	0.09	0.2	0.37	2.95	
**Random effects**:
**Groups**	**Term**	**Std.Dev**.	**Corr**		
item	(Intercept)	0.65		
subj	(Intercept)	2.81			
	logRT	0.45	−0.996	
Number of obs: 11,494, groups: item, 20; subj, 15.					
**Fixed effects**:
	Estimate	Std. Error	*z*-value	Pr (>|z|)	
(Intercept)	−11	0.83	−13	1.5e-38	***
logRT	1.8	0.13	14	6.2e-43	***
n400.fitted	12	1.7	7.2	7.5e-13	***
p300.fitted	−28	2.8	−9.9	2.8e-23	***
logRT:n400.fitted	−2	0.23	−8.7	4.1e-18	***
logRT:p300.fitted	4.6	0.38	12	2.1e-33	***

*Dependent variable is the response accuracy, correspondingly the model family is binomial with a logit link. RT is the total reaction time, i.e., the response tone latency plus the reaction time to that tone. The EEG predictors are the average fitted response extracted from the respective models for each component. Model fit by maximum likelihood using the Laplace approximation and the bobyqa optimizer*.

**Figure 6:**
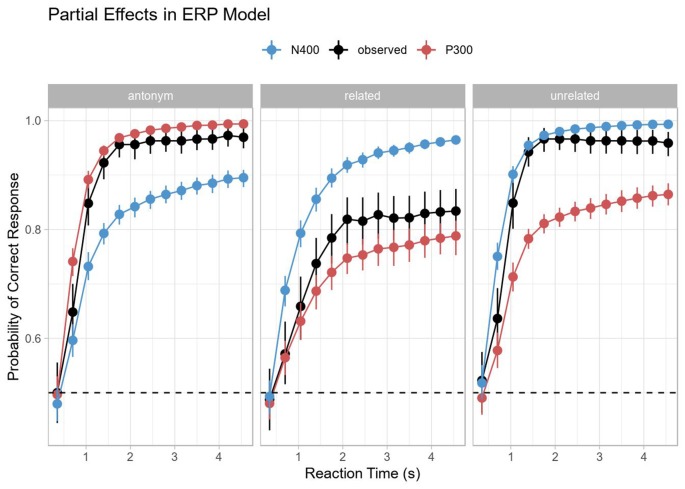
Partial Effects in the ERP model. Note that the P300 predicts performance in the antonym condition and the N400 predicts performance in the unrelated condition. Neither the P300 nor the N400 predicts performance particularly well in the related condition, but the P300 seems to do a slightly better job despite the clear N400 effect and a lack of a clear P300 effect in the ERP data for the same condition. Error bars indicated 95% bootstrap confidence intervals computed on the display (response) scale.

The partial effect plot in Figure 6 shows this most clearly. The curves for each component were obtained by removing the effect for the respective other component (by setting the corresponding predictor to zero using the remef package, Hohenstein and Kliegl, 2015). In the antonym condition, the P300 dominates and this reflects the dominant categorization process for a full prediction match. In the unrelated condition, the N400 dominates and reflects processing the complete prediction mismatch. In the related condition, the N400 is also the dominant effect, but less so, reflecting a mixture of matching (i.e., semantically related) and mismatching features. The partial effects for each individual component, but especially for the N400, make a further prediction for the related condition: both the predicted rate of increase towards terminal accuracy and the terminal accuracy would have been lower than in the unrelated condition. In other words, the largest processing difficulties arise from stimuli that neither completely fulfill predictions nor are clear errors, even though such stimuli do not necessarily elicit the largest ERP components. Thus, this too is in line with the hypothesis sketched above that semantic relatedness interferes with antonym categorization.

Moreover, the main effect for the N400 response in the model reflects an increased probability of correct responses with a decreased N400 amplitude; the accompanying interaction effect with log RT shows that this effect decreases with longer response latencies (see the asymptotic behavior of the N400 curve in Figure 6). This may suggest that the processes underlying the N400 become more decoupled from the categorization process over time, which fits with our assumption that stimulus processing (as reflected in the N400) and categorization states (as reflected in the P300) are connected, yet distinct processes.

Meanwhile, the P300 shows the opposite effect: the main effect of P300 amplitude reflects an initially lower probability of correct response, while its interaction with log RT shows that P300 amplitude is associated with a higher probability of correct response as a function of time. This is compatible with previous research suggesting a decoupling of P300 peak latency and response accuracy at shorter response latencies (see Kutas et al., 1977) and with recent proposals that P300 activity, in general, may reflect the ongoing accumulation of evidence for subsequent decision-making (Twomey et al., 2015).

Overall, this shows that in the antonym design as implemented here, ERP responses to antonyms are indexing categorization dynamics with little influence from N400 activity, while the reverse holds for the two mismatch conditions. Our results also suggest that N400 and P300 responses show reversed influences on accuracy depending on response latency. With increasing response time, reduced N400 amplitudes predict correct responses to a lesser degree, whereas P300 is a worse predictor for response accuracy at shorter latencies. This suggests that ERP-behavior links inferred from standard RT measures are likely to show variation depending on whether the behaviorally indexed decision point falls in early or late bins on the overall continuum of response times in a given experiment.

## Analysis of Component Overlap in The Eeg Data

Throughout the article, and specifically in our modeling of the SAT response as a function of the average amplitude of the P300 and N400 components, we have assumed that these two components are largely independent, or at least two sides of the coin. Furthermore, while our chosen time windows reduce component overlap, they do not eliminate it. To better understand the relationship between the two components, we take a two-pronged approach, considering both the P300 amplitude as a covariate in predicting the N400 amplitude and a multivariate Bayesian model, which allows for modeling both components simultaneously in a single model. The analysis source code is available on the OSF (see “Data Availability Statement” below).

### Using the P300 Amplitude as a Covariate in the N400 Model

**Figure 7:**
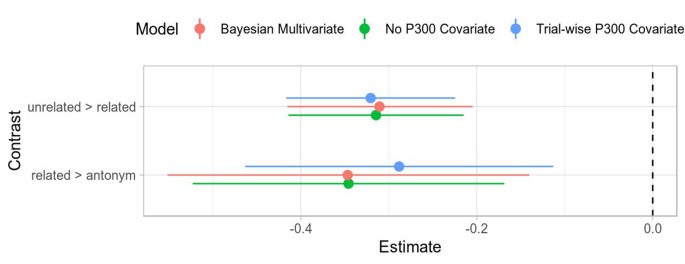
Comparison of coefficient estimates with different overlap corrections for the N400. Uncertainty intervals for the frequentist models are Wald 95% intervals (i.e., twice the standard error). The uncertainty intervals for the Bayesian model is the 95% credible interval. The overall estimates are all quite close and within each other’s uncertainty intervals. The Bayesian model suggests slightly more uncertainty than the frequentist model. Note that all estimates are on the standard deviation scale.

The simplest way to address component overlap is by including the scaled trial-wise P300 amplitude as a predictor for the N400 amplitude as a main effect, which significantly improved model fit. Subsequent extension of this model by including all interaction terms did not significantly improve fit and so we prefer the simpler, more parsimonious model. Interestingly, neither the overall pattern of effects nor their numerical estimates changed much (see Figure 7), indicating that the P300 amplitude is an additive effect or offset for the N400 amplitude. The lack of an interaction effect and similar estimates for the other contrasts suggest that there is some component overlap in the N400 time window, but that the observed effects are independent of the effects in the P300 time window.

**Figure 8:**
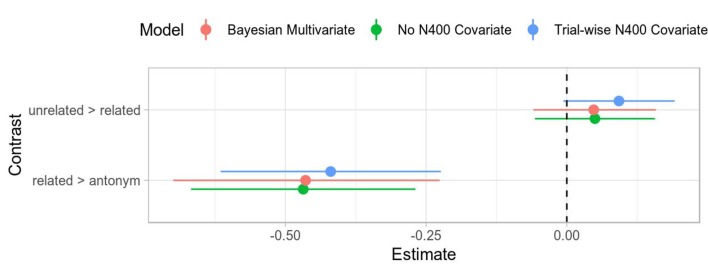
Comparison of coefficient estimates with different overlap corrections for the P300. Uncertainty intervals for the frequentist models are Wald 95% intervals (i.e., twice the standard error). The uncertainty intervals for the Bayesian model is the 95% credible interval. The overall estimates are all quite close and within each other’s uncertainty intervals. The Bayesian model suggests slightly more uncertainty than the frequentist model. Note that all estimates are on the standard deviation scale.

Although it may seem backward in time, we can also repeat this covariate analysis for the P300. This would accommodate for a rising N400 already occurring and overlapping with the P300 in the P300 time window. We again find that the overall model fit is better but that the effect is additive and does not greatly change our contrasts of interest (see Figure 8).

### Bayesian Multivariate Model

Including the trial-wise P300 amplitude in the model for the N400 shows that our N400 effects are not strongly influenced by the preceding P300 (even if the total amplitude in the N400 time window is). However, we can go beyond treating the P300 as an offset for the N400 and jointly model both effects using multivariate Bayesian mixed-effects models with brms (v2.7.0) and Stan (v2.18.2; Bürkner, 2017, 2018; Stan Development Team, 2018). In simple terms, these models can be thought of distinct, simultaneous models that nonetheless inform each other, much in the same way that different groups in a mixed-effect model inform each other *via* partial pooling. This information sharing across submodels furthermore allows for examining covariance between shared predictors for multiple dependent variables and more directly reflects the intertwined nature of the data. In other words, it allows examining how effects are related across different dependent variables. This is similar to structural equation modeling; indeed, it is possible to compute many structural equation models this way.

Given that the frequentist results suggest that including the P300 amplitude as a covariate does not greatly impact our effect estimates in the N400 time window, we omit it from the multivariate model for computational efficiency. As in the EEG analysis above, we use the mean voltage in the baseline window as well as condition as fixed-effect predictors. Our random effects are identical to the analysis above (see “Analysis and Results” section), but with an additional correlation level for the by-item and by-subject effects across dependent measures. Our dependent measures are simultaneously the P300 and N400 responses. All variables are coded and transformed as above.

No priors were set on the random effects beyond the default, which yields point estimates for the random effects comparable to lme4. For the fixed effects, a normal prior with mean of 0 and standard deviation of 2 was used. This is a lightly regularizing prior, equal to the assumption that most effects are small (68% are less than two standard deviations in size) and nearly all are not large (95% are less than four standard deviations in size). This is analogous to weakly-penalized ridge (L2-regularized) regression in frequentist estimation.

The model was fit using Markov Chain Monte Carlo and the No-U-Turn-Sampler (Homan and Gelman, 2014), a self-tuning variant of Hamiltonian Monte Carlo. For all parameters, the Gelman-Rubin statistic Rhat was equal to 1.0 and the number of effective samples exceeded 4,000; for the condition contrasts, the number of effective samples exceeded 7,500. A full model summary can be found in the Supplementary Materials.

The correlation of the by-subject random effects across response variables was not distinguishable from zero (the credible interval crossed zero for all pairwise correlations). This suggests that between-subject variation in the P300 response is not noticeably correlated with the between-subject variation in the N400 response. The correlation of residuals between the different response variables was small but non zero (credible interval: 0.05–0.13). This suggests that there is shared residual variation in both components that is not captured by our predictors.

**Figure 9:**
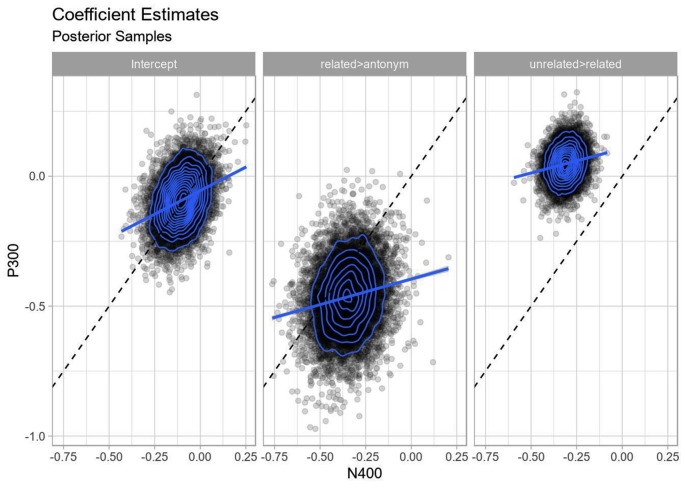
Correlation of fixed effects. Each point represents a posterior sample from the Bayesian multivariate model, blue rings indicate two-dimensional density estimates. The dashed line indicates the line with unit slope through the origin, while the solid lines indicate regression lines through the samples. Strong positive correlation would show itself as the posterior densities forming ovals stretched parallel to the dashed line as well as the parallel regression lines being parallel to the dashed line, while perpendicular axes would indicate strong negative correlation. Note that neither holds: there is no strong correlation between the estimates of the coefficients for P300 and the N400.

The correlation for the fixed effects between components was always positive, but generally small (Pearson correlation of 0.12–0.31; see also Figure 9). This corresponds to some component overlap—a positive deflection from a P300 will shift the basis for the negative deflection for N400 in the positive direction, much like the additive offset behavior in the frequentist model—but does not correspond to completely dependent components, where we would expect stronger collinearity.

Finally, the overall estimates for all effects are similar to the univariate analyses above, although with a larger uncertainty for the related > antonym contrast, reflecting a somewhat larger uncertainty in component-wise amplitude differences between strongly P300-evoking and the strongly N400-evoking conditions (see Figures 7, 8).

Taken together, the frequentist covariate models for each component and the Bayesian multivariate model provide converging evidence for the observed effects for each component being independent of each other and not profoundly distorted by temporal overlap.

## General Discussion

The present article revisited a long-standing issue in the EEG literature on language processing, namely the relationship between multidimensional, time-sensitive electrophysiological data and unidimensional, time-insensitive behavioral data. We hypothesized that previous investigations on this issue faced two methodological challenges: the inherent ambiguity in offline RT measures, conflating response speed, accuracy and different kinds of online processes, and the temporal (and topographical) component overlap of endogenous ERPs such as the N400 and P300. In dealing with the first challenge, we proposed that using time-sensitive behavioral measures such as the SAT paradigm may moderate interpretative ambiguity of RT measures resulting from only observing a single snapshot of completed processing. As for the second challenge, we proposed that cross-method mixed-effects models may be a feasible solution. We examined these issues with the antonym paradigm that has yielded conflicting ERP and behavioral results as well as a strong overlap of N400 and P300 responses to target words.

In terms of the interpretive ambiguity of standard RT measures, we found that time-sensitive behavioral measures can provide more insightful data. Specifically, the SAT data showed that unexpected non-antonym targets that were related to the correct antonym exhibited lowest terminal accuracy and slowest increase in accuracy. This pattern of results is compatible with the view that semantic relatedness of an unexpected sentence completion hinders categorization by sharing semantic features with the expected antonym or, equivalently, overlapping in along a different categorization axis (e.g., for the word pair *black*-*yellow* this would be the feature of being a color term). In line with the interference assumption proposed for semantic relatedness, we did not find a significant difference between antonyms and unrelated non-antonyms in their terminal accuracy nor the trajectory towards it. This clearly contrasts with the results reported previously where the unrelated condition was processed significantly different than the antonym condition (Bentin, 1987; Roehm et al., 2007; Federmeier et al., 2010). Given that RT measures using a single button press constitute just one data point on a SAT curve, one may speculate that the contrast observed previously fell within an RT range in which the differences between the two conditions were most pronounced, while failing to capture dynamic development between earlier and later bins with indistinguishable asymptotes. One way to test this possibility would be to use varying latencies between target word and decision prompts in future ERP experiments on antonym processing, i.e., merging a single-response SAT design (e.g., McElree et al., 2003) with EEG collection. If done carefully, this would also allow for the separation of stimulus- and response-locked components, an aspect that, due to experimental setup, we could not address in our treatment of the P300 (and N400) responses. One open question for further research is whether response-locked components may be a better predictor of SAT responses, thereby also revealing whether it makes a difference to categorize a prediction match or mismatch.

Our modeling of SAT responses as a function of EEG activity lends further support to the hypothesis that standard RT measures may be measuring different spots along the SAT curve (also across conditions), which is not under experimental control. We found interactive effects for response time and ERP activity as predictors of response accuracy. Importantly, while reductions in N400 amplitude were a better predictor for response accuracy at shorter latencies, the reversed pattern held for the P300. Surely, any inference as to which ERP component influences response accuracy obtained with standard RT measures will depend on where on the hypothesized SAT curve that RT data point will be positioned. As argued above, this can be accounted for by systematically sampling the latencies between RT measures and target processing or, as already proposed by others, by modeling accuracies as a function of response time (e.g., Davidson and Martin, 2013). Finally, our modeling approach also attests to the feasibility that ERP responses in one sample predict behavioral SAT responses in another, and may therefore be particularly suitable for experimental designs, where the specifics of the single-response SAT procedure appear impossible to be combined with EEG recordings for practical reasons (e.g., due to the higher number of experimental trials needed to compute a robust signal, resulting in an excessive number of experimental sessions). In general, the modeling technique proposed here also applies to combining EEG with further behavioral methods, such as eye-tracking or skin conductance, that may necessitate partly different experimental designs than EEG setups to guarantee internal validity.

Regarding ERP component overlap in time, we hypothesized that the N400 and P300 responses during linguistic categorization show related, yet distinguishable processes. Specifically, we conjectured that the N400 would be more sensitive to processing stimulus properties relevant for categorization (including linguistic fit, see Bornkessel-Schlesewsky and Schlesewsky, 2019), while the P300 indexes the dynamics of the categorization process itself (O’Connell et al., 2012; Twomey et al., 2015). Component overlap is a notorious problem in interpreting ERP patterns, as it makes it extremely difficult to determine whether amplitude modulations in a given component under study are the result of offsets introduced by an adjacent component (additive component overlap), reflective of modulations within a given component or a mixture of the two (multiplicative component overlap). One way to address this problem in the case of overlapping N400 and P300 responses is to deploy the attested sensitivity of the P300 to task variation and associated attention orientation. That is, naturalistic tasks (e.g., reading or listening for comprehension) or tasks that direct participants’ attention away from stimulus properties used for linguistic categorization help reduce P300 overlap (e.g., Roehm et al., 2007; Haupt et al., 2008). Yet, task variation may not always be an option for various reasons, the most obvious one being that categorization itself is of interest. The present article takes the extreme version of the opposite end of task variation: using a behavioral measure to help disentangle components. Our choice of behavioral task and stimulus paradigm elicits a strong categorization response (P300) independent of a response to the congruency and fit of the stimulus (N400). This results in ERP effects that we can separate statistically and which provide a useful basis for decomposing and understanding processing time-courses as exhibited behaviorally in the SAT paradigm. In other words, understanding the perception-action loop can be better understood when we manipulate both perception and action.

In summary, the current experiments and analyses strongly suggest that combining EEG with time-sensitive behavioral measures from SAT designs enriches our understanding of both ERPs elicited by language input and the resulting behavioral performance in categorization tasks. The SAT data suggest that, in the antonym paradigm, N400 priming effects due to semantic relatedness do not affect behavioral performance, unless they impact negatively on categorization, whereas categorization processes clearly dominate response behavior. As a consequence, the current SAT data can be integrated more readily with explanations of the possible cognitive functions of the N400 (stimulus-related processes) and the P300 (categorization dynamics).

In our modeling approach, we have restricted ourselves to two components and their temporal overlap to demonstrate the feasibility of this type of cross-method analyses. There are several possibilities of how our modeling approach can be extended in future research. First, recall that our data sets are based on a stimulus paradigm that yields near-perfect cloze probability for the predicted target word. An obvious application is to test the current approach with experimental designs inducing a broader range of cloze probability values to measure predictability. Second, our modeling approach can be applied to other types of component mixtures as well. This includes not only topographic overlap of distinct ERPs, but also temporal overlap of the N400 and the ensuing late positivity. Throughout the present article, for instance, we have argued that the positivity in response to non-antonyms indexes eventual categorization for prediction mismatches, hence is also a P300 with a latency shift. Follow-up studies could test to what extent late positivities in other experimental designs overlap with or are independent of the N400, thereby also further testing assumptions on the nature of the late positivity (see Leckey and Federmeier, 2019).

## Conclusion

We presented here a novel application of modern statistical approaches to better understand the complex interaction between behavior and electrophysiology and more generally between offline and online measures. We demonstrated a general technique for combining data from multiple methods, resulting in a novel decomposition of competing neural processes underlying behavior. Subsequently, we used a combination of techniques to disentangle two classically entwined ERP effects, the P300 and N400, with potential applications to other component mixtures. To see the dynamics of processing in its full depth, we must examine distinct measures together, much in the same way that depth perception arises from combining two distinct perspectives. Only in the combination of perception and action do we see the full loop and thus, by closing the perception-action loop, we learn more about both perception and action.

## Data Availability

The datasets generated for this study (preprocessed EEG data and SAT data) along with analysis source code on the Open Science Framework (OSF) will be made available upon publication of this manuscript. For review purposes, these may be viewed at https://osf.io/75r6t/?view_only=b1c45caff34a48558b580e8a7202cfe7.

## Ethics Statement

Experiment 1 was conducted in the XLinc Lab at the University of Cologne. The protocol for ERP experiments conducted in the lab is approved by the Ethics Committee of the German Society of Linguistics (DGfS; #2016-09-160914). Experiment 2 was not accompanied by an ethics vote but was conducted in line with national and institutional guidelines. Specifically, behavioral non-invasive experiments with healthy young adults (between 18–65 years) do not require one as long as they pose no risk or physical/emotional burden to participants and as long as participants are debriefed after participation as specified by the rules of the German Research Foundation (DFG; https://www.dfg.de/foerderung/faq/geistes_sozialwissenschaften/, archived page on 27 Feb 2019 available at https://web.archive.org/web/20190227214057/https://www.dfg.de/foerderung/faq/geistes_sozialwissenschaften/).

## Author Contributions

FK designed the experimental protocol and ran the experiments. PA analyzed the data. FK and PA wrote the manuscript.

## Conflict of Interest Statement

The authors declare that the research was conducted in the absence of any commercial or financial relationships that could be construed as a potential conflict of interest.

## Footnotes

^1^A reviewer noted that in non-SAT lexical-decision and semantic-relatedness experiments, participants typically respond faster with positive/yes answers than negative/no answers and expressed concern that comparing yes and no responses is thus an unfair comparison. To address this concern, we note that the RT difference between the response polarity exactly follows the experimental manipulation, i.e., is not typically separable from the effect of condition. Moreover, this is line with current computational and psychological theory. Verifying a word is much faster than rejecting a nonword because the word can be accepted as soon as a match is found in the mental lexicon, while an exhaustive search is necessary for a nonword. Or in terms of activation: the baseline activation of a real word is much higher than a nonword and so processing is easier and faster. Second, in our case, the difference between yes and no responses is somewhat separable from the effect of condition because there are multiple conditions which require a no response (all but the actual antonym condition) and which nonetheless differ in their SAT curves. That is, unrelated non-antonyms are different from related ones in speed and accuracy, even though both require participants to give a No response in order to correctly perform the task. This suggests that the differences between conditions are at least in part due to the experimental manipulation and not the yes/no distinction. Of course, having multiple No conditions and only one Yes condition results in a lack of balance. In the overall experiment, this is eliminated *via* the filler sentences. For the analysis of the critical sentences, this is not problematic for the statistical methods used, as the dependent variable was encoded as accuracy and not response polarity. Finally, the nature of RT within the multiple response-SAT paradigm, where the response latencies are largely determined by the experimenter and not the participant, should preclude any such differences. Indeed, this is born out in the data, with no differences between positive and negative responses (see “Data Availability Statement” below for links to the data and scripts on OSF).
