# Supplementary material for: Speed-Accuracy Tradeoffs in Brain and Behavior: Testing the Independence of P300 and N400 Related Processes in Behavioral Responses to Sentence Categorization
Source: Front Hum Neurosci. 2019 Aug 27;13:285. doi: 10.3389/fnhum.2019.00285 (PMC6718734; doi:10.3389/fnhum.2019.00285)
Supplement: Supplementary file 1 [file Data_Sheet_1.PDF]

Supplementary Materials

Table of Contents

Frequentist Covariate Models 2
  N400 2
    Comparison of Additive and Multiplicative Models 2
    Comparison of Basic and Overlap Models 2
    Summary of Best Additive Overlap Model 2
  P300 3
    Comparison of Additive and Multiplicative Models 3
    Comparison of Basic and Overlap Models 3
    Summary of Best Additive Overlap Model 3
Bayesian Modelling of ERP data 4
  Model Summary 5
  Correlations across Response Variables 7
    Random Effects 7
    Residuals 7
    Fixed effects 7
  Markov Chains 8
Session Information 16

Frequentist Covariate Models

N400

Comparison of Additive and Multiplicative Models

	Df	AIC	BIC	logLik	deviance	Chisq	Chi Df	Pr(>Chisq)
Additive Model	15	6959	7049	-3465	6929			
Interaction with condition	17	6960	7061	-3463	6926	4	2	0.17
All interactions	20	6964	7083	-3462	6924	2	3	0.54

Comparison of Basic and Overlap Models

	Df	AIC	BIC	logLik	deviance	Chisq	Chi Df	Pr(>Chisq)
Basic Model	14	7002	7086	-3487	6974			
P300 covariate	15	6959	7049	-3465	6929	45	1	< 0.001

Summary of Best Additive Overlap Model

```
## Linear mixed model fit by maximum likelihood  ['lmerMod']
## Formula: scale(n400) ~ 1 + scale(p300) + scale(baseline) * cond + (1 +
cond | subj) + (1 | item)
##    Data: eeg.cp
## Control: lmerControl(optimizer = "bobyqa", calc.derivs = FALSE)
##
##      AIC      BIC   logLik deviance df.resid
##   6959.3   7048.8  -3464.6   6929.3     2883
##
## Scaled residuals:
##     Min      1Q  Median      3Q     Max
## -3.0298 -0.6538  0.0309  0.6448  5.0190
##
## Random effects:
##  Groups   Name                    Variance Std.Dev. Corr
##  item     (Intercept)             0.01707  0.1306
##  subj     (Intercept)             0.08434  0.2904
##           condrelated > antonym   0.13423  0.3664   -0.35
##           condunrelated > related 0.01242  0.1114    0.52  0.62
##  Residual                         0.60441  0.7774
## Number of obs: 2898, groups:  item, 80; subj, 20
##
## Fixed effects:
```

```
##                                          Estimate Std. Error t value
## (Intercept)                             -0.125417   0.068343  -1.835
## scale(p300)                              0.109787   0.016175   6.788
## scale(baseline)                         -0.178207   0.017319 -10.290
## condrelated > antonym                   -0.495278   0.089720  -5.520
## condunrelated > related                 -0.325061   0.048261  -6.735
## scale(baseline):condrelated > antonym    0.050135   0.038768   1.293
## scale(baseline):condunrelated > related  0.002496   0.043327   0.058
##
## Correlation of Fixed Effects:
##             (Intr) s(300) scl(b) cndr>a cndn>r s():>a
## scale(p300)  0.021
## scale(bsln) -0.014 -0.078
## cndrltd>ant -0.282  0.085 -0.009
## cndnrltd>rl  0.254 -0.014 -0.084  0.092
## scl(bsl):>a -0.005 -0.043  0.308  0.013 -0.018
## scl(bsl):>r -0.020  0.009 -0.015 -0.004 -0.069 -0.562
```

P300

Comparison of Additive and Multiplicative Models

	Df	AIC	BIC	logLik	deviance	Chisq	Chi Df	Pr(>Chisq)
Additive Model	15	7696	7785	-3833	7666			
Interaction with condition	17	7696	7798	-3831	7662	4	0.17	0.61
All interactions	20	7698	7817	-3829	7658	4	0.24	0.62

Comparison of Basic and Overlap Models

	Df	AIC	BIC	logLik	deviance	Chisq	Chi Df	Pr(>Chisq)
Basic Model	14	7743	7827	-3858	7715			
N400 covariate	15	7996	7785	-3833	7666	50	1	< 0.001

Summary of Best Additive Overlap Model

```
## Linear mixed model fit by maximum likelihood  ['lmerMod']
## Formula: scale(p300) ~ 1 + scale(n400) + scale(baseline) * cond + (1 +
cond | subj) + (1 | item)
##    Data: eeg.cp
## Control: lmerControl(optimizer = "bobyqa", calc.derivs = FALSE)
##
##      AIC      BIC   logLik deviance df.resid
##   7695.6   7785.1  -3832.8   7665.6     2883
```

```
##
## Scaled residuals:
##     Min      1Q  Median      3Q     Max
## -4.3940 -0.6410 -0.0249  0.6495  3.5622
##
## Random effects:
##  Groups   Name                      Variance Std.Dev. Corr
##  item     (Intercept)               0.007636 0.08739
##  subj     (Intercept)               0.052825 0.22984
##           condrelated > antonym     0.191659 0.43779  -0.07
##           condunrelated > related 0.007160 0.08462  -0.98  0.25
##  Residual                           0.791877 0.88987
## Number of obs: 2898, groups:  item, 80; subj, 20
##
## Fixed effects:
##                                     Estimate Std. Error t value
## (Intercept)                         -0.06911    0.05528  -1.250
## scale(n400)                          0.14848    0.02080   7.138
## scale(baseline)                      0.11383    0.01990   5.719
## condrelated > antonym               -0.38928    0.10674  -3.647
## condunrelated > related              0.09003    0.05134   1.754
## scale(baseline):condrelated > antonym    0.09800    0.04508   2.174
## scale(baseline):condunrelated > related -0.02019    0.04923  -0.410
##
## Correlation of Fixed Effects:
##             (Intr) s(400) scl(b) cndr>a cndn>r s():>a
## scale(n400)  0.051
## scale(bsln) -0.007  0.172
## cndrltd>ant -0.020  0.107  0.016
## cndnrltd>rl -0.331  0.128 -0.063 -0.105
## scl(bsl):>a -0.007 -0.029  0.324  0.012 -0.024
## scl(bsl):>r -0.027 -0.002 -0.073 -0.005 -0.069 -0.585
```

Bayesian Modelling of ERP data

Model Summary

```
##  Family: MV(gaussian, gaussian)
##   Links: mu = identity; sigma = identity
##          mu = identity; sigma = identity
## Formula: scale(p300) ~ 1 + scale(baseline) * cond + (1 + cond | p | subj) + (1 | q | item)
##          scale(n400) ~ 1 + scale(baseline) * cond + (1 + cond | p | subj) + (1 | q | item)
##    Data: eeg.cp (Number of observations: 2898)
## Samples: 4 chains, each with iter = 10000; warmup = 1000; thin = 3;
##          total post-warmup samples = 12000
##
## Group-Level Effects:
## ~item (Number of levels: 80)
##                                              Estimate Est.Error l-95% CI u-95% CI Eff.Sample Rhat
## sd(scalep300_Intercept)                          0.12      0.02     0.07     0.16       9931 1.00
## sd(scalen400_Intercept)                          0.15      0.02     0.11     0.19      10083 1.00
## cor(scalep300_Intercept,scalen400_Intercept)     0.84      0.13     0.52     0.99       7054 1.00
##
## ~subj (Number of levels: 20)
##                                                                     Estimate Est.Error l-95% CI u-95% CI Eff.Sample Rhat
## sd(scalep300_Intercept)                                                 0.28      0.05     0.19     0.41       9218 1.00
## sd(scalep300_condrelated>antonym)                                       0.54      0.11     0.36     0.79       9991 1.00
## sd(scalep300_condunrelated>related)                                     0.12      0.07     0.01     0.28       7900 1.00
## sd(scalen400_Intercept)                                                 0.35      0.06     0.25     0.49      11047 1.00
## sd(scalen400_condrelated>antonym)                                       0.46      0.09     0.30     0.67      11377 1.00
## sd(scalen400_condunrelated>related)                                     0.13      0.06     0.01     0.26       9248 1.00
## cor(scalep300_Intercept,scalep300_condrelated>antonym)                 -0.11      0.22    -0.51     0.32       9033 1.00
## cor(scalep300_Intercept,scalep300_condunrelated>related)               -0.28      0.32    -0.80     0.44      13086 1.00
## cor(scalep300_condrelated>antonym,scalep300_condunrelated>related)      0.08      0.33    -0.56     0.69      12515 1.00
## cor(scalep300_Intercept,scalen400_Intercept)                            0.33      0.20    -0.10     0.67      11928 1.00
## cor(scalep300_condrelated>antonym,scalen400_Intercept)                 -0.32      0.20    -0.67     0.10      10882 1.00
## cor(scalep300_condunrelated>related,scalen400_Intercept)               -0.08      0.32    -0.67     0.56       5739 1.00
## cor(scalep300_Intercept,scalen400_condrelated>antonym)                 -0.10      0.22    -0.52     0.34      12131 1.00
## cor(scalep300_condrelated>antonym,scalen400_condrelated>antonym)        0.17      0.22    -0.29     0.58      11243 1.00
## cor(scalep300_condunrelated>related,scalen400_condrelated>antonym)      0.23      0.33    -0.47     0.78       5027 1.00
## cor(scalen400_Intercept,scalen400_condrelated>antonym)                 -0.25      0.21    -0.63     0.19      12173 1.00
## cor(scalep300_Intercept,scalen400_condunrelated>related)               -0.25      0.30    -0.76     0.39      12157 1.00
## cor(scalep300_condrelated>antonym,scalen400_condunrelated>related)      0.12      0.30    -0.50     0.67      12328 1.00
## cor(scalep300_condunrelated>related,scalen400_condunrelated>related)    0.30      0.35    -0.48     0.85       7305 1.00
## cor(scalen400_Intercept,scalen400_condunrelated>related)                0.20      0.29    -0.42     0.71      12156 1.00
```

```
## cor(scalen400_condrelated>antonym,scalen400_condunrelated>related)       0.27      0.29    -0.35     0.77      11270 1.00
##
## Population-Level Effects:
##                                              Estimate Est.Error l-95% CI u-95% CI Eff.Sample Rhat
## scalep300_Intercept                             -0.09      0.07    -0.23     0.05       8097 1.00
## scalen400_Intercept                             -0.14      0.08    -0.29     0.03      10574 1.00
## scalep300_scalebaseline                          0.09      0.02     0.05     0.13      12146 1.00
## scalep300_condrelated>antonym                   -0.47      0.13    -0.72    -0.21       8365 1.00
## scalep300_condunrelated>related                  0.04      0.06    -0.07     0.16      12253 1.00
## scalep300_scalebaseline:condrelated>antonym      0.11      0.05     0.02     0.20      12468 1.00
## scalep300_scalebaseline:condunrelated>related   -0.03      0.05    -0.13     0.07      11903 1.00
## scalen400_scalebaseline                         -0.17      0.02    -0.20    -0.13      13731 1.00
## scalen400_condrelated>antonym                   -0.55      0.11    -0.77    -0.33      11015 1.00
## scalen400_condunrelated>related                 -0.32      0.05    -0.42    -0.21      11969 1.00
## scalen400_scalebaseline:condrelated>antonym      0.07      0.04    -0.01     0.15      12327 1.00
## scalen400_scalebaseline:condunrelated>related   -0.01      0.05    -0.10     0.08      11951 1.00
##
## Family Specific Parameters:
##                 Estimate Est.Error l-95% CI u-95% CI Eff.Sample Rhat
## sigma_scalep300     0.90      0.01     0.87     0.92      13693 1.00
## sigma_scalen400     0.78      0.01     0.76     0.80      12957 1.00
##
## Residual Correlations:
##                             Estimate Est.Error l-95% CI u-95% CI Eff.Sample Rhat
## rescor(scalep300,scalen400)     0.11      0.02     0.08     0.15      13432 1.00
##
## Samples were drawn using sampling(NUTS). For each parameter, Eff.Sample
## is a crude measure of effective sample size, and Rhat is the potential
## scale reduction factor on split chains (at convergence, Rhat = 1).
```

Correlations across Response Variables

Random Effects

```
sign(VarCorr(eeg.mvmodel)[["subj"]]$cor[1:3,"Q2.5",4:6]) !=
sign(VarCorr(eeg.mvmodel)[["subj"]]$cor[1:3,"Q97.5",4:6])
```

	**scalen400_Intercept**	**scalen400_condantonym**	**scalen400_condrelated**
scalep300_Intercept	TRUE	TRUE	TRUE
scalep300_condantonym	TRUE	TRUE	TRUE
scalep300_condrelated	TRUE	TRUE	TRUE

Residuals

```
VarCorr(eeg.mvmodel)[["residual__"]]$cor["scalen400",,"scalep300"]
##   Estimate  Est.Error       Q2.5      Q97.5
## 0.11348675 0.01869162 0.07663292 0.14996596
hdi(VarCorr(eeg.mvmodel,summary=FALSE)[["residual__"]]$cor[,"scalen400","scalep300
"])
##      lower      upper
## 0.07816224 0.15119537
## attr(,"credMass")
## [1] 0.95
```

Fixed effects

The correlations in the fixed-effects are small but significant (visible in the plots being somewhat elliptical, but with the major (long) axis being largely parallel to the x-axis) – this is line with component overlap.

	**r**	**p**
Intercept	0.33	< 0.001
related>antonym	0.17	< 0.001
unrelated>related	0.20	< 0.001

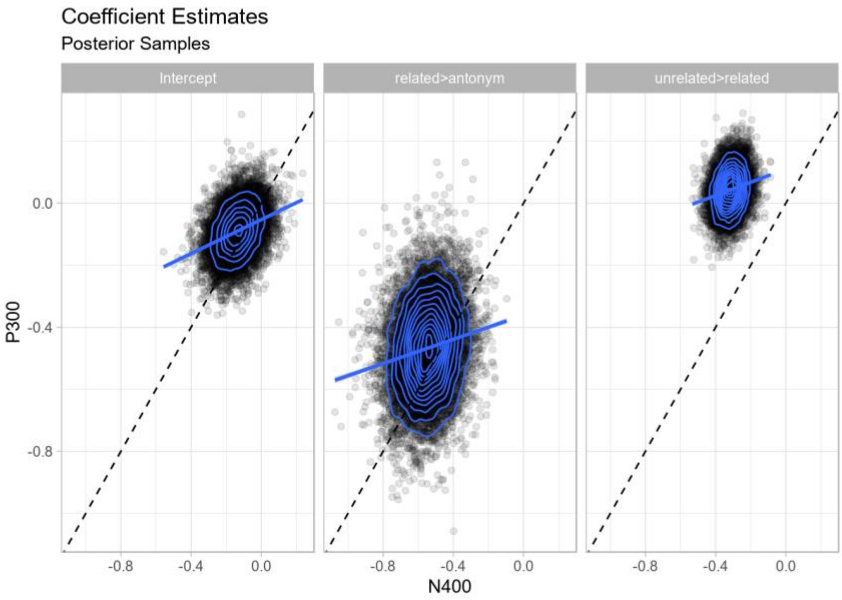

Markov Chains

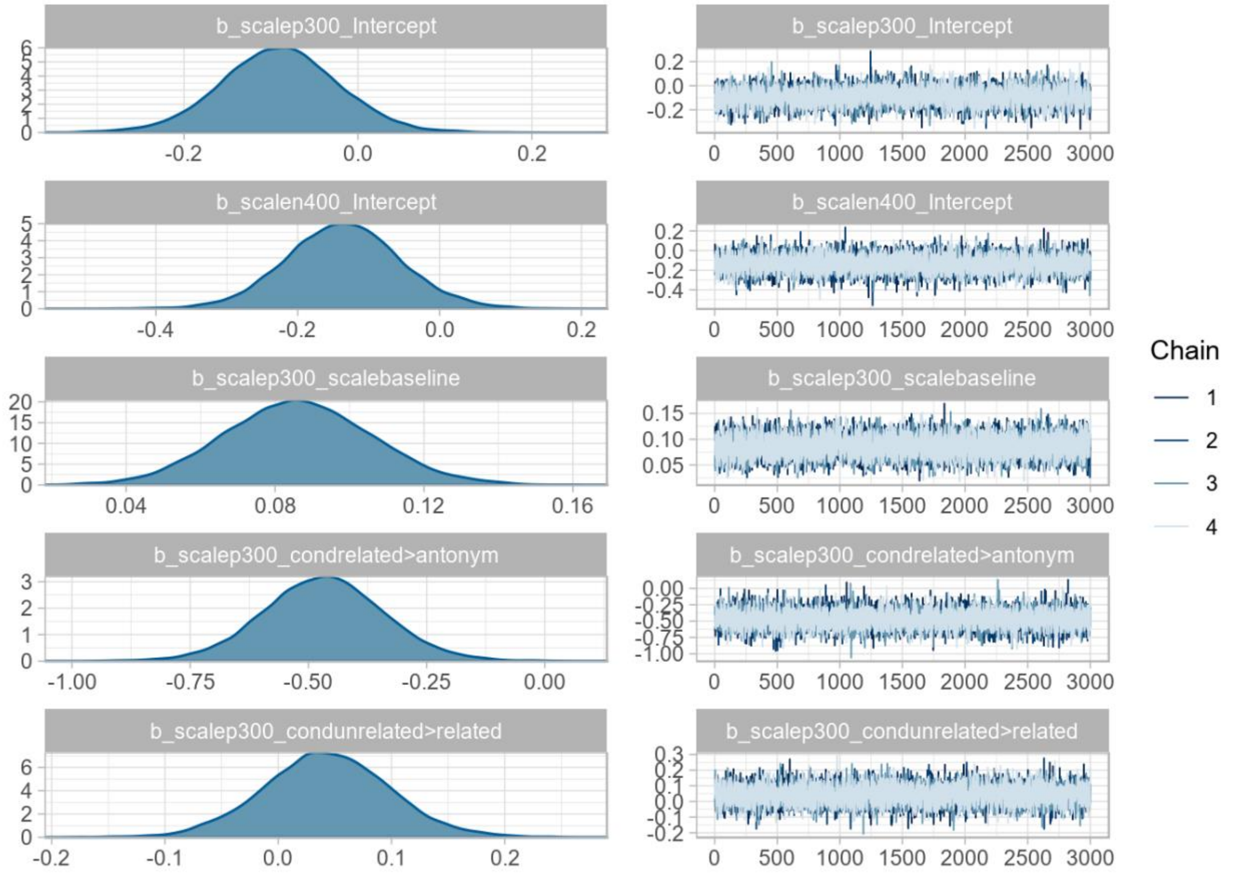

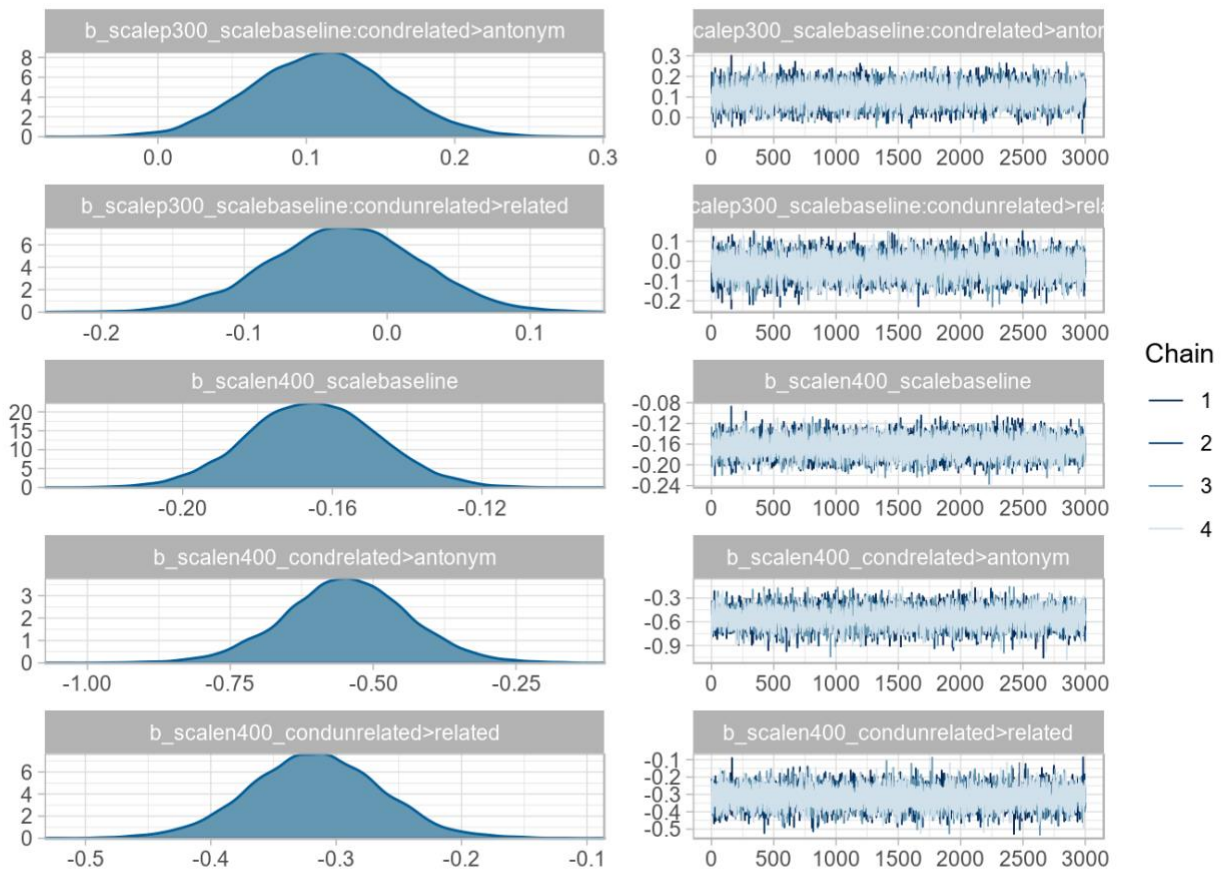

b_scalep300_scalebaseline:condrelated>antonym
b_scalep300_scalebaseline:condunrelated>related
b_scalen400_scalebaseline
b_scalen400_condrelated>antonym
b_scalen400_condunrelated>related
Chain
1
2
3
4

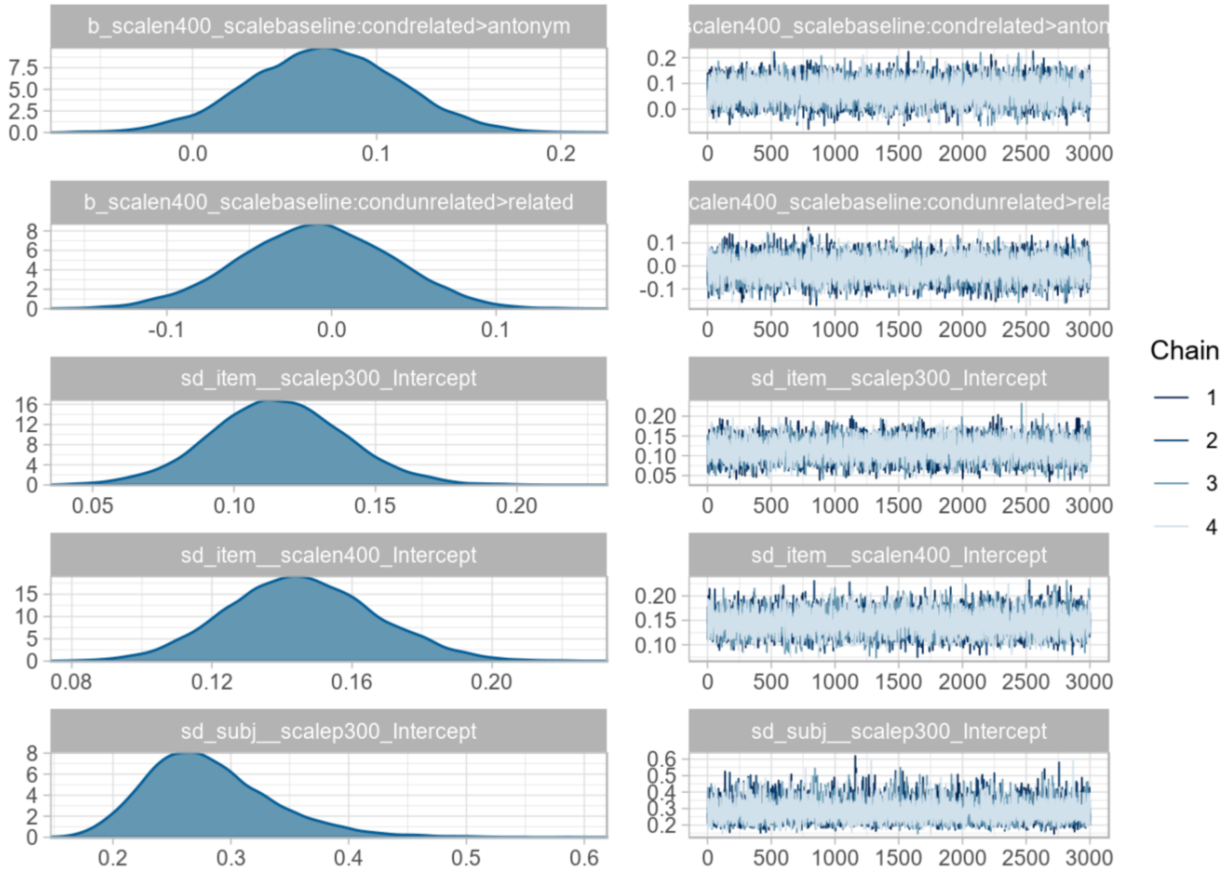
b_scalen400_scalebaseline:condrelated>antonym
scalen400_scalebaseline:condrelated>anton
b_scalen400_scalebaseline:condunrelated>related
scalen400_scalebaseline:condunrelated>rela
sd_item__scalep300_Intercept
sd_item__scalep300_Intercept
sd_item__scalen400_Intercept
sd_item__scalen400_Intercept
sd_subj__scalep300_Intercept
sd_subj__scalep300_Intercept
Chain
1
2
3
4

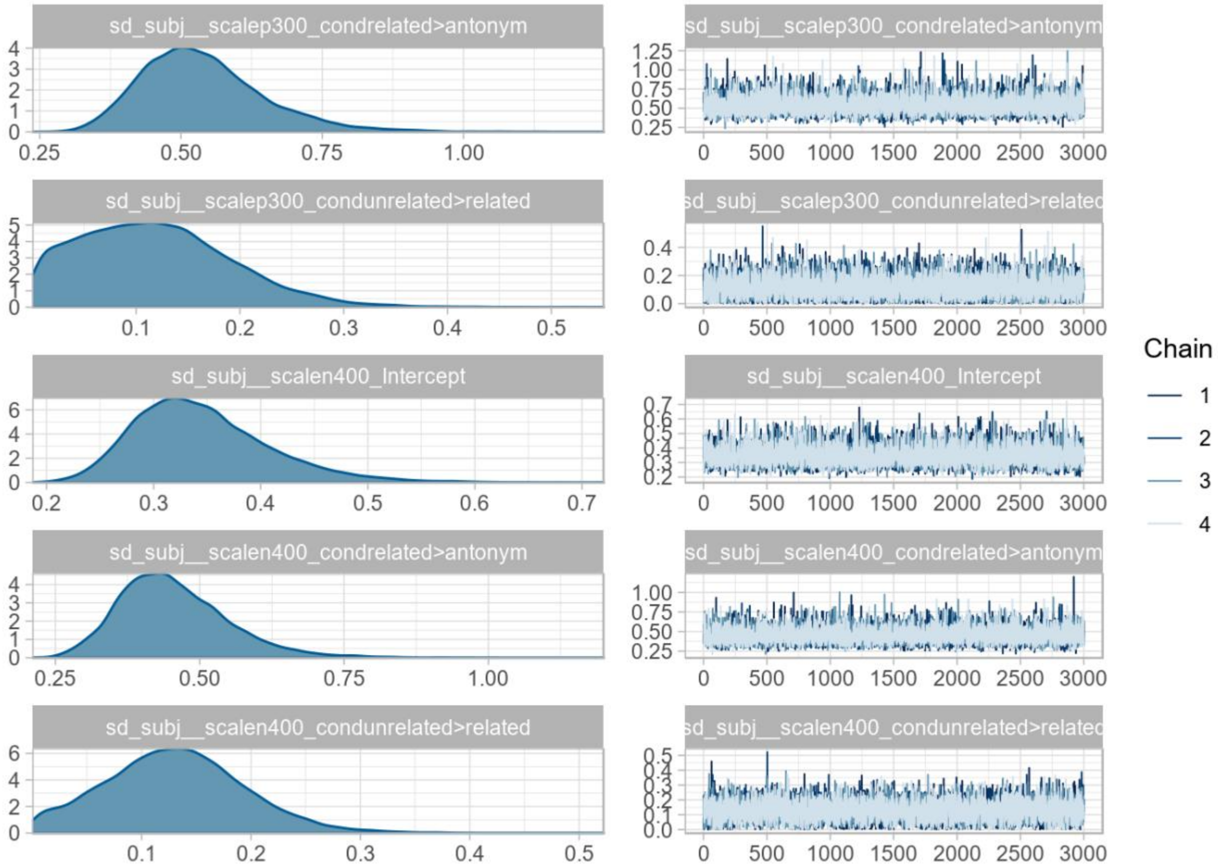
sd_subj__scalep300_condrelated>antonym
sd_subj__scalep300_condunrelated>related
sd_subj__scalen400_Intercept
sd_subj__scalen400_condrelated>antonym
sd_subj__scalen400_condunrelated>related
Chain
1
2
3
4

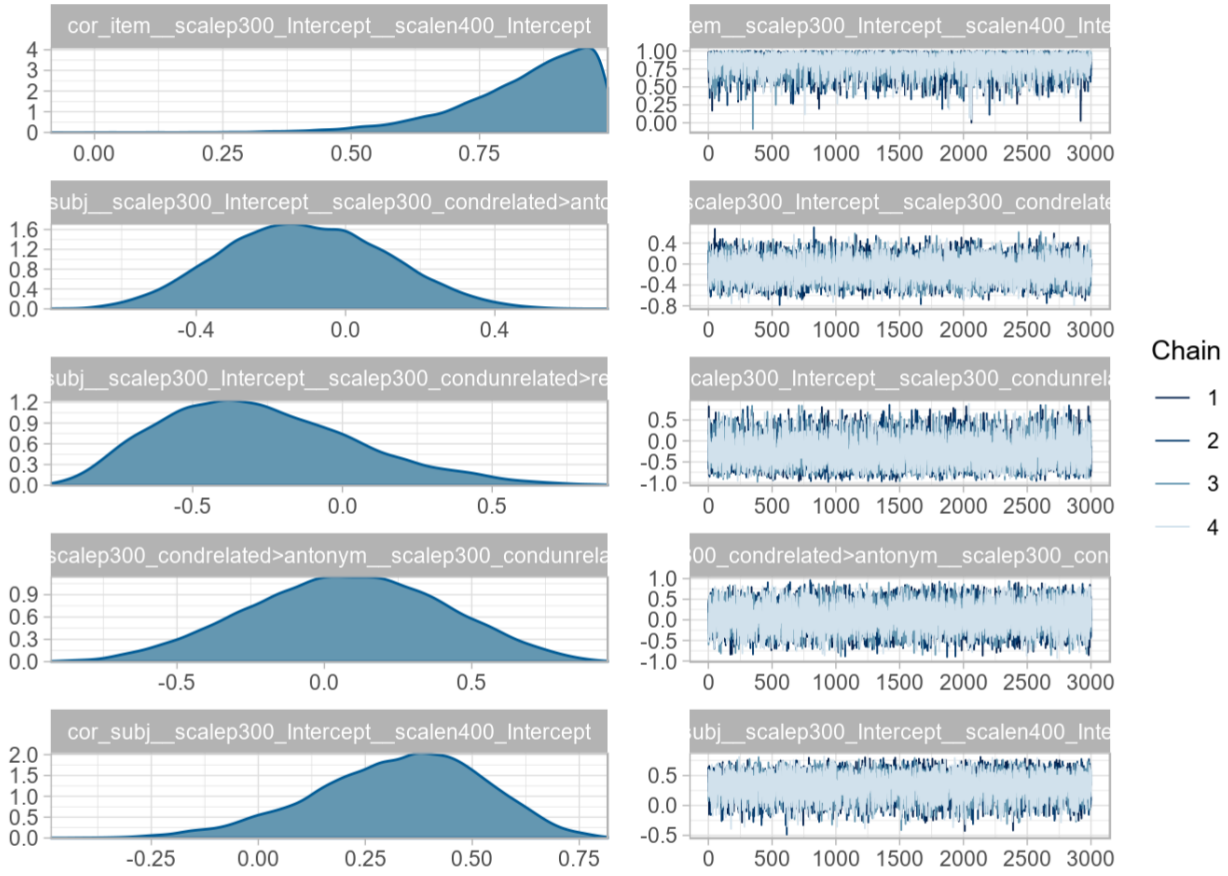
cor_item__scalep300_Intercept__scalen400_Intercept
subj__scalep300_Intercept__scalep300_condrelated>anto
subj__scalep300_Intercept__scalep300_condunrelated>re
scalep300_condrelated>antonym__scalep300_condunrela
cor_subj__scalep300_Intercept__scalen400_Intercept
Chain
1
2
3
4

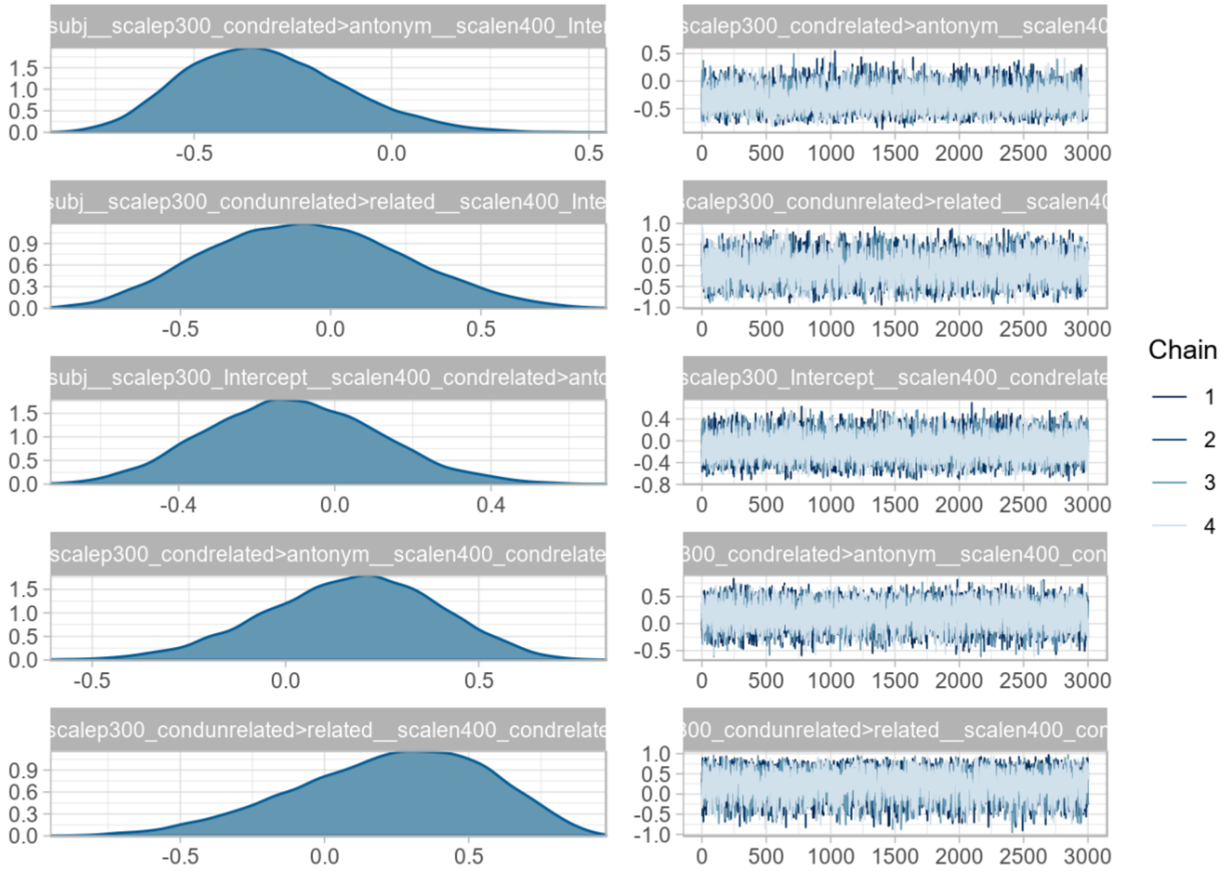

subj__scalep300_condrelated>antonym__scalen400_Inte
scalep300_condrelated>antonym__scalen40
subj__scalep300_condunrelated>related__scalen400_Inte
scalep300_condunrelated>related__scalen40
subj__scalep300_Intercept__scalen400_condrelated>ant
scalep300_Intercept__scalen400_condrelate
scalep300_condrelated>antonym__scalen400_condrelate
300_condrelated>antonym__scalen400_con
scalep300_condunrelated>related__scalen400_condrelate
300_condunrelated>related__scalen400_con
Chain
1
2
3
4

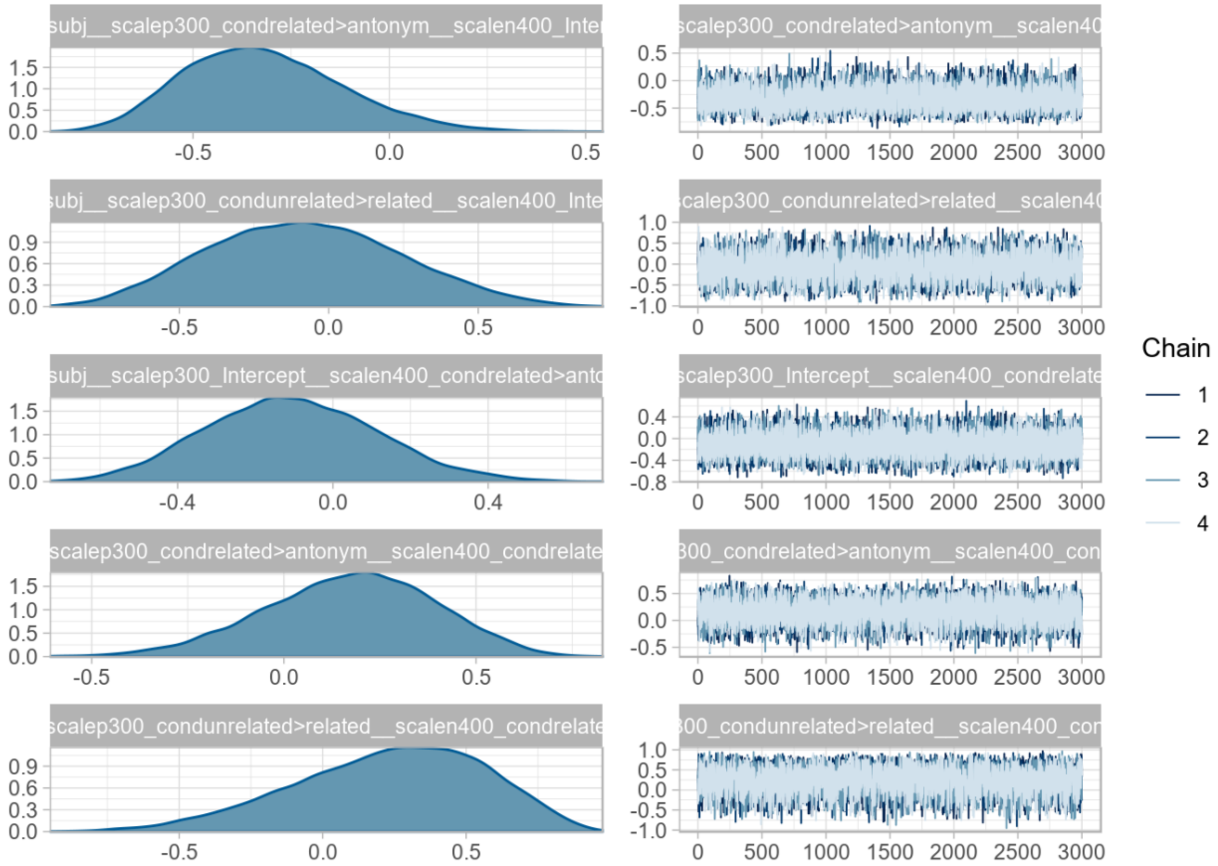

subj__scalep300_condrelated>antonym__scalen400_Inte
scalep300_condrelated>antonym__scalen40
subj__scalep300_condunrelated>related__scalen400_Inte
scalep300_condunrelated>related__scalen40
subj__scalep300_Intercept__scalen400_condrelated>anto
scalep300_Intercept__scalen400_condrelate
scalep300_condrelated>antonym__scalen400_condrelate
300_condrelated>antonym__scalen400_con
scalep300_condunrelated>related__scalen400_condrelate
300_condunrelated>related__scalen400_con
Chain
1
2
3
4

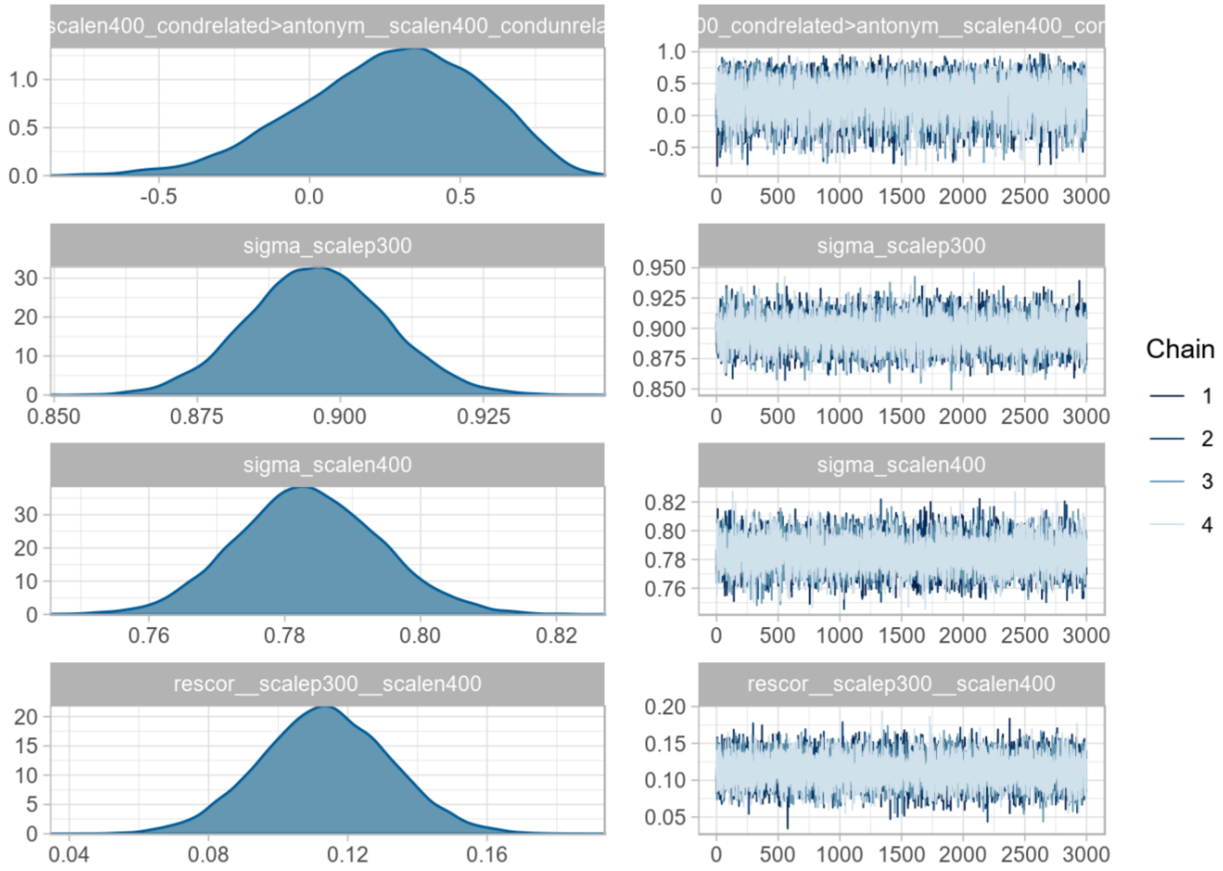

scalen400_condrelated>antonym__scalen400_condunrela
00_condrelated>antonym__scalen400_con
sigma_scalep300
sigma_scalen400
rescor__scalep300__scalen400
Chain
1
2
3
4

Session Information

```
sessionInfo()
## R version 3.5.0 (2018-04-23)
## Platform: x86_64-suse-linux-gnu (64-bit)
## Running under: openSUSE Leap 15.0
##
## Matrix products: default
## BLAS: /usr/lib64/R/lib/libRblas.so
## LAPACK: /usr/lib64/R/lib/libRlapack.so
##
## locale:
##  [1] LC_CTYPE=en_GB.UTF-8       LC_NUMERIC=C               LC_TIME=en_AU.UTF-8
LC_COLLATE=en_GB.UTF-8     LC_MONETARY=nl_NL.UTF-8
##  [6] LC_MESSAGES=en_GB.UTF-8    LC_PAPER=nl_NL.UTF-8       LC_NAME=C                  LC_ADDRESS=C
LC_TELEPHONE=C
## [11] LC_MEASUREMENT=nl_NL.UTF-8 LC_IDENTIFICATION=C
##
## attached base packages:
## [1] stats     graphics  grDevices utils     datasets  methods   base
##
## other attached packages:
##  [1] psycho_0.4.91         neuropsychology_0.5.0 MASS_7.3-51.1         HDInterval_0.2.0
emmeans_1.3.2         magrittr_1.5          lmerOut_0.5
##  [8] latex2exp_0.4.0       printr_0.1            car_3.0-2             carData_3.0-2
forcats_0.4.0         stringr_1.4.0         dplyr_0.8.1
## [15] purrr_0.3.2           readr_1.3.1           tidyr_0.8.3           tibble_2.1.1
ggplot2_3.1.1         tidyverse_1.2.1       remef_1.0.6.9000
## [22] brms_2.8.0            Rcpp_1.0.1            lme4_1.1-20           Matrix_1.2-15
##
## loaded via a namespace (and not attached):
##   [1] estimability_1.3       wordcloud2_0.2.1       SparseM_1.77           ModelMetrics_1.2.2
lavaan_0.6-3           coda_0.19-2
##   [7] nonnest2_0.5-2         acepack_1.4.1          knitr_1.23             dygraphs_1.1.1.6
data.table_1.12.0      rpart_4.1-13
##  [13] inline_0.3.15          generics_0.0.2         callr_3.2.0            xml2_1.2.0
lubridate_1.7.4        httpuv_1.5.1
##  [19] StanHeaders_2.18.1     assertthat_0.2.1       d3Network_0.5.2.1      gower_0.1.2
xfun_0.7               hms_0.4.2
##  [25] bayesplot_1.6.0        evaluate_0.14          promises_1.0.1         fansi_0.4.0
readxl_1.2.0           igraph_1.2.4.1
##  [31] htmlwidgets_1.3        mcmc_0.9-5             stats4_3.5.0           crosstalk_1.0.0
backports_1.1.4        pbivnorm_0.6.0
##  [37] markdown_0.9           ggcorrplot_0.1.2       MCMCpack_1.4-4         vctrs_0.1.0
quantreg_5.38          abind_1.4-5
##  [43] caret_6.0-81           withr_2.1.2            checkmate_1.9.1        fdrtool_1.2.15
xts_0.11-2             prettyunits_1.0.2
##  [49] mnormt_1.5-5           cluster_2.0.7-1        lazyeval_0.2.2         crayon_1.3.4
recipes_0.1.4          pkgconfig_2.0.2
##  [55] slam_0.1-44            labeling_0.3           nlme_3.1-137           ggm_2.3
nnet_7.3-12            rlang_0.3.4
##  [61] miniUI_0.1.1.1         colourpicker_1.0       MatrixModels_0.4-1     sandwich_2.5-1
modelr_0.1.2           cellranger_1.1.0
##  [67] tcltk_3.5.0            matrixStats_0.54.0     loo_2.1.0              boot_1.3-20
zoo_1.8-5              base64enc_0.1-3
##  [73] whisker_0.3-2          ggridges_0.5.1         processx_3.3.0         rjson_0.2.20
png_0.1-7              jpeg_0.1-8
##  [79] shinystan_2.5.0        scales_1.0.0           plyr_1.8.4             threejs_0.3.1
compiler_3.5.0         rstantools_1.5.1
##  [85] RColorBrewer_1.1-2     cli_1.1.0              lmerTest_3.1-0         pbapply_1.4-0
ps_1.3.0               Brobdingnag_1.2-6
##  [91] htmlTable_1.13.1       Formula_1.2-3          tidyselect_0.2.5       stringi_1.4.3
highr_0.8              yaml_2.2.0
##  [97] latticeExtra_0.6-28    bridgesampling_0.6-0   grid_3.5.0             manipulate_1.0.1
tools_3.5.0            parallel_3.5.0
## [103] rio_0.5.16             rstudioapi_0.10        foreach_1.4.4          foreign_0.8-71
gridExtra_2.3          prodlim_2018.04.18
## [109] BDgraph_2.59           digest_0.6.18          shiny_1.3.2            lava_1.6.4
ppcor_1.1              broom_0.5.2
## [115] BayesFactor_0.9.12-4.2 later_0.8.0            httr_1.4.0             rsconnect_0.8.13
psych_1.8.12           colorspace_1.4-1
```

```
## [121] blavaan_0.3-4          rvest_0.3.2             splines_3.5.0           expm_0.999-3
shinythemes_1.1.2      rstanarm_2.18.2
## [127] MuMIn_1.42.1           xtable_1.8-4            jsonlite_1.6            nloptr_1.2.1
corpcor_1.6.9          timeDate_3043.102
## [133] rstan_2.18.2           glasso_1.10             zeallot_0.1.0           NLP_0.2-0
ipred_0.9-8            nFactors_2.3.3
## [139] R6_2.4.0               tm_0.7-6                Hmisc_4.2-0             pillar_1.4.0
htmltools_0.3.6        mime_0.6
## [145] glue_1.3.1             minqa_1.2.4             DT_0.5                  class_7.3-15
codetools_0.2-16       pkgbuild_1.0.3
## [151] mvtnorm_1.0-10         utf8_1.1.4              lattice_0.20-38         numDeriv_2016.8-1
huge_1.3.2             curl_3.3
## [157] DescTools_0.99.28      gtools_3.8.1            zip_1.0.0               shinyjs_1.0
openxlsx_4.1.0         survival_2.43-3
## [163] CompQuadForm_1.4.3     rmarkdown_1.12          qgraph_1.6.3            munsell_0.5.0
iterators_1.0.10       haven_2.0.0
## [169] reshape2_1.4.3         gtable_0.3.0
```
